# Memo Has a Novel Role in S1P Signaling and Crucial for Vascular Development

**DOI:** 10.1371/journal.pone.0094114

**Published:** 2014-04-08

**Authors:** Shunya Kondo, Alessia Bottos, Jeremy C. Allegood, Regis Masson, Francisca G. Maurer, Christel Genoud, Patrick Kaeser, Andrea Huwiler, Masato Murakami, Sarah Spiegel, Nancy E. Hynes

**Affiliations:** 1 Friedrich Miescher Institute for Biomedical Research, Basel, Switzerland; 2 Department of Biochemistry and Molecular Biology, Virginia Commonwealth University School of Medicine, Richmond, Virginia, United States of America; 3 University of Basel, Basel, Switzerland; 4 Institute of Pharmacology, University of Bern, Bern, Switzerland; 5 Novartis Institutes for Biomedical Research, Basel, Switzerland; University of Geneva, Switzerland

## Abstract

Memo is a conserved protein that was identified as an essential mediator of tumor cell motility induced by receptor tyrosine kinase activation. Here we show that Memo null mouse embryonic fibroblasts (MEFs) are impaired in PDGF-induced migration and this is due to a defect in sphingosine-1-phosphate (S1P) signaling. S1P is a bioactive phospholipid produced in response to multiple stimuli, which regulates many cellular processes. S1P is secreted to the extracellular milieu where it exerts its function by binding a family of G-protein coupled receptors (S1PRs), causing their activation in an autocrine or paracrine manner. The process, termed cell-autonomous S1PR signaling, plays a role in survival and migration. Indeed, PDGF uses cell-autonomous S1PR signaling to promote cell migration; we show here that this S1P pathway requires Memo. Using vascular endothelial cells (HUVECs) with Memo knock-down we show that their survival in conditions of serum-starvation is impaired. Furthermore, Memo loss in HUVECs causes a reduction of junctional VE-cadherin and an increase in sprout formation. Each of these phenotypes is rescued by S1P or S1P agonist addition, showing that Memo also plays an important role in cell-autonomous S1PR signaling in endothelial cells. We also produced conventional and endothelial cell-specific conditional Memo knock-out mouse strains and show that Memo is essential for embryonic development. Starting at E13.5 embryos of both strains display bleeding and other vascular problems, some of the phenotypes that have been described in mouse strains lacking S1PRs. The essential role of Memo in embryonic vascular development may be due in part to alterations in S1P signaling. Taken together our results show that Memo has a novel role in the S1P pathway and that Memo is needed to promote cell-autonomous S1PR activation.

## Introduction

Our group identified Memo (mediator of ErbB2-driven cell motility) as a 34 kDa protein essential for robust breast cancer cell motility in response to activation of several receptor tyrosine kinases (RTKs)[1]. Memo knock-down (KD) tumor cells showed decreased migration following treatment with epidermal growth factor (EGF), heregulin (HRG) or fibroblast growth factor (FGF)[1], [2]. Memo is encoded by a gene that is found in all kingdoms of life; Memo's protein sequence is highly conserved[3]. In *S. cerevisiae* we have found that Memo has a role in invasive growth, suggesting that a function in cell motility/invasion is conserved across species. In mice, Memo is widely expressed in most adult organs[4]. Using a conditional Memo knock-out (KO) strain we have recently shown that Memo loss leads to reduced life-span accompanied by elevated serum levels of vitamin D and calcium and other phenotypes[4]. The exact *in vivo* physiological role of Memo is still unknown, however, the dramatic effects of Memo loss suggest that in addition to its role in migration, Memo might be involved in other essential physiological processes.

Here we show that Memo null mouse embryonic fibroblasts (MEFs) isolated from *Memo* fl/fl embryos[4] are also impaired in platelet-derived growth factor (PDGF)-induced migration. Interestingly, we found that this is due to a defect in sphingosine-1-phosphate (S1P) signaling in MEFs lacking Memo. S1P is a bioactive sphingolipid metabolite that is produced within cells through phosphorylation of sphingosine, in a reaction catalyzed by two sphingosine kinases (SphK1 and −2)[5]–[7]. S1P has a crucial role in many physiological processes including vascular development and lymphocyte trafficking[7], [8]. S1P is secreted to the extracellular milieu by several transporters[9] where it exerts its biological function by binding a family of five G-protein coupled receptors, S1PR1-5, that are widely expressed in most cells and tissues. Many stimuli activate SphKs leading to the production and secretion of S1P, which activates S1PRs in an autocrine or paracrine manner. This process of cell-autonomous S1PR activation, also called ‘inside-out’ signaling, plays a role in, e.g., survival and migration[6], [10]. Indeed, growth factors such as PDGF, use autonomous signaling of the SphK1/S1PR1 axis to promote cell migration[11]–[15].

S1P has an essential role in stabilizing blood vessels during development[16], [17] and is important for endothelial proliferation, migration, angiogenesis and survival[18]. This prompted us to examine Memo's role in endothelial cells. Using vascular endothelial cells (HUVECs) with Memo KD we show that survival is impaired in conditions of low S1P, following serum-starvation and, importantly, this phenotype can be rescued by S1P addition, suggesting that Memo is required for cell-autonomous signaling. We also produced conventional and endothelial cell-specific conditional Memo KO mouse strains and show here that Memo is essential for embryonic development. Starting at E13.5 embryos of both stains display bleeding and other vascular problems, some of the phenotypes that have been described in mouse strains lacking SphKs or S1PRs[19]–[21]. Taken together our results suggest that Memo has a novel role in the S1P pathway, namely in promoting cell-autonomous S1PR signaling.

## Materials and Methods

### Ethics Statement

Animals were housed in a 12/12 hour light/dark cycle with food and water provided ad libitum, and all animal samples were obtained after sacrificing animals with CO2 inhalation. All animal experiments were performed according to Ethical Principles and Guidelines for Experiments on Animals (3^rd^ edition 2005, Switzerland) and approved by the Institutional Animal Care and Use Committee, the FMI Animal Committee, following approval by the Cantonal Veterinary Office Basel-Stadt (Permit number 2286).

### Materials

D-erythro-sphingosine-1-phosphate (S1P) and D-erythro-sphingosine were purchased from Enzo Life Sciences (Farmingdale, NY). Recombinant human platelet-derived growth factor-BB (PDGF-BB), recombinant human vascular endothelial growth factor 165 (VEGF_165_) and mouse anti-His tag antibody were sourced from R&D systems (Minneapolis, MN). SEW2871 and fatty acid-free bovine serum albumin (BSA) was purchased from Sigma (St. Louis, MO). VPC23019 and (R)-W146 were obtained from Avanti Polar Lipids (Alabaster, AL). VPC96091 was kindly provided by Kevin R. Lynch (University of Virginia). [γ-^32^P] ATP (3000 Ci/mmol) was obtained from Hartmann Analytic (Braunschweig, Germany). Mouse anti-smooth muscle actin (SMA) antibody (1A4) and anti-mouse CD31 rat antibody (MEC13.3) was purchased from Sigma and BD biosciences (San Diego, CA), respectively. Rabbit anti-VE cadherin antibody (#sc-28644) and mouse anti-myc antibody (9E10) were obtained from Santa Cruz Biotechnology. Alexa-Fluor 546 conjugated goat anti-rat antibody, Alexa-Fluor 488 conjugated goat anti-rabbit antibody and mouse anti-V5 antibody were obtained from Invitrogen (Carlsbad, CA). Rabbit anti-phospho-ERK (p42/44 MAPK) antibody and rabbit anti-phospho-VEGFR2 antibody (19A10) were obtained from Cell Signaling Technology (Boston, MA). Mouse anti-α-tubulin antibody (DM1A) was purchased from NeoMarkers (Fremont, CA). The anti-Memo monoclonal antibody was produced in house as described previously[2] and recognizes human and mouse Memo.

### Cell culture, transfection, virus production and infection

Mouse embryonic fibroblasts (MEFs) were generated from E13.5 *Memo* fl/fl embryos, maintained in Dulbecco's modified Eagle's medium (DMEM) supplemented with 10% fetal bovine serum (FBS) (Sigma) and spontaneously immortalized by continuous passaging. Human umbilical vein endothelial cells (HUVECs) were purchased from PromoCell (Germany) and maintained in Endothelial Cell Growth Medium (PromoCell). HUVECs of passage 5-8 were used for all assays.

For deletion of *Memo* in MEFs, a retrovirus was produced using the vector pMSCV-CreERT^2^-puromycin (a gift from Patrick Matthias (FMI, Basel, Switzerland)) following the protocol described in Yamaguchi et al.[22]. *Memo* fl/fl MEFs were infected with the retrovirus and cultures were exposed 3 days to tamoxifen to activate Cre and delete *Memo*.

To generate stable control and Memo KD in HUVECs the following vectors were used. The shLacZ control vector was produced cloning the sequences 5′-CCGGGCGGCTGCCGGAATTTACCTTCTCGAGGGTAAATTCCGGCAGCCGCTTTTT-3′/5′-AATTAAAAAGCGGCTGCCGGAATTTACCCTCGAGAAGGTAAATTCCGGCAGCCGC-3 into the pLKO.1-puro plasmid. Stocks of pLKO.1-puro vectors containing different shRNA sequences targeting Memo were purchased from Sigma (MISSION shRNA libraryTRCN0000122895 (sh1) and TRCN0000122898 (sh2)).

For all lentiviral preparations, HEK293T cells were transiently transfected with 8 μg pLKO.1-puro construct, 0.4 μg HDM-tat16, 0.4 μg HDM-HgPM2, 0.4 μg pRC-CMV-RaII and 0.8 μg HDM-VSV-G using polyethylenimine (PEI) (Polysciences Inc., Warrington, PA, USA) and incubated for 16 hours (h) at 37°C, at which point the media was changed. Media containing viruses were collected 72 h post-transfection, filter sterilized, and stored at −80°C. Cells were infected over-night at 37°C with lentiviral particles at a multiplicity of infection (MOI) of 2 in the presence of 8 μg/ml polybrene (Sigma) for 24 h. The media was then changed and the cells were incubated another 24 h at 37°C. Successfully infected cells were selected using 500 ng/mL puromycin (Sigma).

To analyze interaction between Memo and SphK1, HEK293T cells were transiently transfected with pcDNA3.1-myc-Memo[1] and/or pcDNA3.1-V5-SphK1[15] using PEI and cultured for 48 h before harvesting.

### Immunoprecipitation, His pull-down and western blotting

Cells were lysed in NP-40 lysis buffer and subjected to immunoprecipitation as described previously[2]. For His pull-down assay, purified Memo or myc-Memo[23] was incubated with purified His-SphK1 (a gift from Doriano Fabbro (NIBR, Basel, Switzerland)) for 16 h at 4°C in pull-down assay buffer (50 mM Tris, pH 7.5, 100 mM NaCl, 1 mM EDTA, 0.1% NP-40, 10% glycerol, 20 mM imidazole, 1 mM phenylmethylsulfonyl fluoride, 10 μg/ml leupeptin, and 10 μg/ml aprotinin). The protein mixture was incubated with Ni-NTA agarose (Qiagen) for 90 minutes at 4°C, washed three times with pull-down assay buffer and the bound proteins were eluted from the agarose. Protein lysates were immunoblotted as described previously[2].

### Cell migration assay

The transwell migration of cells was determined by modified Boyden chamber assays. Briefly, both sides of an 8 μm-pore migration chamber (24-well format: BD Biosciences, San Diego, CA) were coated with 25 μg/ml rat tail Type I collagen for MEFs or 5 μg/ml fibronectin for HUVECs (both from Roche, Basel, Switzerland). Cells were starved in migration medium (MEFs - overnight in DMEM containing 0.1% fatty acid-free BSA; HUVECs- 4 h in MEM alpha containing 0.1% fatty acid-free BSA) then harvested into the same medium, plated onto the top well and allowed to migrate toward the lower well filled with migration medium containing S1P, PDGF or VEGF. After 16 h for MEFs or 4 h for HUVECs, cells migrated to the lower side of the chamber were fixed with 4% PFA and stained with 0.1% crystal violet. Images were taken at five different areas per well using an Axiovert 200 microscope (Carl Zeiss, Göttingen, Germany) and the number of cells was counted. In experiments with VPC96191 or VPC23019, harvested cells were incubated with them for 60 min at 37°C prior to plating and the same concentration of VPC96191 or VPC23019 was put in the lower wells.

### Quantitative real-time PCR (qPCR)

Total RNA was extracted from cells using the RNeasy Mini Kit (Qiagen) and used to synthesize cDNA by reversed transcriprion with Ready-to-go You-Prime First-Strand Beads (GE Healthcare, Little Chalfont, UK) using oligos-dT(15) primers (Promega, Fitchburg, WI). Quantitative real-time PCR was carried out using specific primers listed in Table S1 with StepOne Real-Time PCR System instrument and software (Applied Biosystems, Carlsbad, CA). For each primer pair, a calibration curve was drawn and used for the calculations. Expression values were normalized to the value of mouse actin or human glyceraldehyde 3-phosphate dehydrogenase (GAPDH).

### Sphingosine kinase assay

SphK1 activity was measured according to Olivera et al.[24] with modifications. Briefly, cells were harvested with the SphK assay buffer (20 mM Tris, pH 7.5, 20% glycerol, 1 mM EDTA, 1 mM phenylmethylsulfonyl fluoride, 1 mM dithiothreitol, 10 μM MgCl_2_, 5 mM sodium orthovanadate, 15 mM NaF, 10 μg/ml leupeptin, and 10 μg/ml aprotinin) and freeze-thawed. Lysates were centrifuged at 700 × g for 10 min at 4°C to remove the insoluble fraction and the supernatants were then centrifuged at 100,000 × g for 60 min at 4°C to obtain the cytosolic fractions as supernatants. Cytoplasmic extracts (10 μg protein) were incubated for 30 min at 37°C in SphK assay buffer in the presence of 50 μM D-erythro sphingosine, [γ-^32^P] ATP (1 mM, 0.5 μCi), 10 mM MgCl_2_ and 0.25% Triton X-100. Lipids were extracted by adding 800 μl chloroform/methanol/HCl (100∶200∶1, v/v), vortexing 1 min, adding 240 μl chloroform and 240 μl 2 M KCl, vortexing 5 min, and centrifuging at 4,000 × g for 2 min. The lower organic phase was dried, resuspended in chloroform/methanol/HCl (100∶200∶1, v/v) and separated by TLC on silica gel 60 (Merck, Germany) with chloroform/acetone/methanol/acetic acid/H_2_O (10∶4∶3∶2∶1, v/v). Radioactive bands were visualized and quantified using an imager, Typhoon 9400 (Amersham Biosciences, Little Chalfont, UK).

### Measurement of S1P levels

Cell culture media were harvested on ice, mixed immediately with 1/20 volume of inhibitor cocktail (300 mM Tris-HCl pH 7.5 containing 40 mM Na_2_VO_4_, 80 mM pyrophosphate, 100 mM NaF, 20 mM deoxypyridoxine, 800 mM glycerophosphate) and frozen at −80°C. Cells were harvested into ice-cold methanol and kept at −80°C. Embryos were freshly frozen at −80°C. Mouse plasma was mixed with 10 times volume of ice-cold methanol and kept at −80°C. Lipids were extracted and the level of S1P was measured by LC-ECI-MS/MS analysis as described previously[25].

### Proliferation and apoptosis assays

HUVECs were plated on 6-well plates and cultured in Endothelial Cell Growth Medium (full media) for 48 h. After washing twice with PBS, the cells were cultured in full media or serum-free alpha MEM with or without S1P or VPC23019. After 24 h, cells attached to the dish were harvested and counted using the Vi-CELL counter (Beckman Coulter). For apoptosis assays, HUVECs were plated on 96-well plates and cultured in full media for 48 h. After washing twice with PBS, the cells were cultured for another 24 h with serum-free alpha MEM with or without S1P or VPC23019. The ratio of apoptotic cells was determined with the YO-PRO apoptosis assay kit (Invitrogen)[26].

### *In situ* hybridization

The 1.15 kb CDS/3′-UTR fragment of mouse Memo cDNA (NCBI Reference Sequence NM_133771.2, nucleotides 347–1493) was PCR amplified and used as a template for generating riboprobes. Digoxigenin-labeled antisense and sense (control) riboprobes were prepared using the DIG RNA Labeling Kit (Roche, Basel, Switzerland). Mouse embryos were fixed in 4% PFA in PBS, and embedded in paraffin. Hybridization of the sections (5 μm thick) was performed with the Discovery XT Staining Module (Ventana Medica Systems S.A.). Hybridized probe-signals were visualized using DIG Nucleic Acid Detection Kit (Roche) and counterstained with Methyl green.

### Generation of Memo mutant mice

We generated conventional and conditional Memo KO mice by gene targeting. For this, we cloned a targeting construct containing a 5′ homology arm, three loxP recombination sites, exon 2 (E2), a FRT-flanked Pgk-Neomycin resistance (NEO) cassette, and a 3′ homology arm (Figure S4A). The targeting construct was linearized and introduced in 129/Ola embryonic stem (ES) cells by electroporation. An ES clone that had undergone homologous recombination was selected and confirmed by PCR and Southern blot analysis. To generate conventional Memo KO mice, the mutant ES clone containing the targeted allele was transiently transfected with the vector expressing cre recombinase (pCMV-CRE; gift from Patrick Matthias, FMI, Basel, Switzerland) to delete the floxed fragment (Figure S4A). An ES clone containing the deleted allele was selected and used to establish chimeric mice. These mice were further bred as *Memo* +/− mice. Conditional Memo KO mice were generated previously[4]. For this, chimeric mice were established using the mutant ES clone containing the targeted allele (Figure S4A), crossed with FLP deleter transgenic mice (gift from Silvia Arber, FMI, Basel, Switzerland) to delete the NEO cassette and further bred as *Memo* fl/+.

Both lines were maintained in the background of four times backcrossed to C57BL/6JRccHsd (Stock number 43) from Harlan Laboratory (Netherland). Genotyping of these lines was performed using primer F (5′-CCCTCTCATCTGGCTTGGTA-3′) and primer R (5′-GCTCGATATGCTCACAAAGG-3′) that recognize the sites indicated in Figure S4A. To produce Memo endothelial cell-specific Memo KO (ECKO) mice, Memo floxed mice were crossed with Tie2Cre transgenic mice in a C57BL/6 background[27] (kindly provided by Tatiana Petrova, ISREC, Lausanne). Conversion of the floxed allele into the deleted allele in the presence of the Tie2Cre allele was confirmed (Figure S5A, lane 3).

### Immunohistochemical analysis

For immunostaining of sections, embryos were fixed in 4% PFA in PBS and embedded in paraffin. Sections (3 μm thick) were stained with the Discovery XT Staining Module (Ventana Medica Systems S.A.). For whole-mount immunostaining, yolk sacs were fixed in 4% PFA in PBS, washed in PBS and incubated with blocking solution (1% skim milk, 0.1% Triton X-100 in PBS). Staining was performed by incubating tissues with anti-CD31 rat antibody in blocking solution, followed by incubation with Alexa-Fluor 546 conjugated anti-rat antibody in blocking solution.

### Electron microscopy

Forelimbs were taken from embryos and fixed overnight at 4°C in 2% PFA and 2% glutaraldehyde in 0.1 M Na-cacodylate buffer (pH 7.4). They were then rinsed for 3×5 min with 0.1 M Na-cacodylate buffer (pH 7.4) and postfixed with 0.1 M Na-cacodylate buffer containing 1.5% potassium ferrocyanide and 1% osmium tetroxide for 30 min, immediately followed by treatment in 1% osmium tetroxide in ddH_2_O for 30 min. To enhance contrast, after 5 rinses in ddH_2_O, forelimbs were stained *en bloc* with 1% aqueous uranyl acetate for 30 min. After dehydration with ethanol, samples were rinsed in propylene oxide and embedded in Embed812 resin (EMS). Blocks were trimmed perpendicular to the finger buds, and ultrathin sections (30–50 nm) were prepared with a Leica Ultracut EM UC7, collected on Formvar coated copper slot grids, and stained with uranyl acetate and Reynold's lead. Images were acquired on a CM10 (FEI, Eindhoven) at 80 kV equipped with a side-mounted digital camera (Veleta, Olympus).

### Immunofluorescent staining

HUVECs were grown to confluency, washed twice with PBS and cultured for another 6 h in full media (Endothelial Cell Growth Medium) or serum-free alpha MEM with or without S1P or VPC23019. Cells were then fixed in 4% PFA in PBS (+) (PBS containing 0.5 mM Mg^++^ and 1 mM Ca^++^) for 5 min at room temperature, permeabilized in 0.2% Triton X-100 in PBS (+) for 8 min at 4°C, and incubated with rabbit anti-VE cadherin antibody in PBS (+) containing 5% skim milk overnight at 4°C. After washing 3 times in PBS (+), the slides were incubated with Alexa-Fluor 488 conjugated goat anti-rabbit antibody for 1 h at room temperature, washed 3 times in PBS (+) and mounted in ProLong Gold Antifade Reagent with DAPI (Invitrogen). Images were taken using LSM700 confocal microscope (Carl Zeiss).

### HUVECs cellular spheroid sprouting assay

HUVECs were harvested and resuspended in Endothelial Cell Basal Medium (EBM) (PromoCell) containing 20% Methocel and 20% charcoal stripped fetal bovine serum (cs-FBS) (Invitrogen) at a density of 4 cells/μl. 800 cells (200 μl)/well were seeded into non-adherent round-bottom 96-wells and cultured overnight to allow spheroid formation. Spheroids (40 spheroids per experimental point) were collected by centrifugation at 1,200 rpm for 5 min and resuspended in 200 μl of 60% Methocel, 40% cs-FBS, 40 ng/ml VEGF165 and 3 U/mL heparin (Calbiochem). In some experiments, SEW2871, VPC23019 and W146 were added to the spheroid suspension. In parallel, the collagen solution was prepared with rat tail Type I collagen (Roche) to give a final concentration of 1.4 mg/ml on ice. The spheroid suspensions were mixed 1∶1 (v/v) with the collagen solution, rapidly transferred into 48-well plates and incubated at 37°C with 5% CO_2_. After 36 h, spheroids were fixed with 4% formaldehyde and imaged with Axiovert200 (Carl Zeiss). Quantification was performed by counting the number of sprouts and measuring the cumulative sprout length per spheroid.

### Statistical analysis

Statistical significance was determined by unpaired two-tailed Student's t test.

## Results

### PDGF induced migration requires Memo to mediate cell-autonomous S1PR signaling

**Figure 1:**
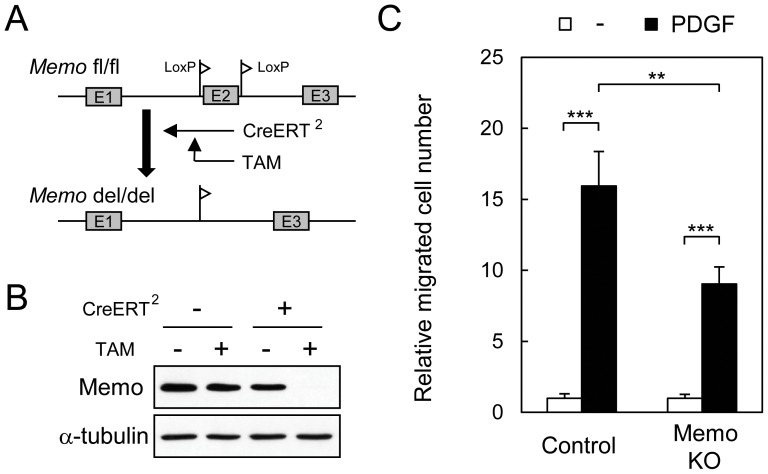
Memo KO fibroblasts have defects in PDGF-induced cell migration. **A**, Diagram for conditional Memo deletion in mouse embryonic fibroblasts (MEFs). Immortalized *Memo* fl/fl MEFs were infected with a CreERT^2^-expressing retrovirus then treated with tamoxifen (TAM). **B**, Western analyses for Memo levels in lysates from cells subjected to the indicated treatments; α-tubulin is the loading control. CreERT^2^-expressing *Memo* fl/fl MEFs without or with TAM treatment were used as control and Memo knock-out (KO) MEFs, respectively. **C**, Transwell cell migration of control and Memo KO MEFs was induced by PDGF (5 ng/ml) in serum-free media. Data were normalized to the average value for basal migration in serum-free media and are presented as means ± S.D. of five individual wells. Similar results were obtained in three independent experiments. **, *p*<0.01; ***, *p*<0.001

Control and Memo KO mouse embryonic fibroblasts (MEFs) were used to explore migration and signaling pathways. For these experiments we used MEFs prepared from *Memo* fl/fl embryos[4]. Memo was deleted by infecting the cells with a CreERT^2^-containing retrovirus, which allows tamoxifen (TAM) inducible Memo deletion (Figure 1A). This system allows a comparison between control and Memo KO MEFs from the same origin. Memo protein was no longer detectable in *Memo* fl/fl MEFs 3 days following TAM addition (Figure 1B). Treatment of control MEFs with PDGF induced robust motility; in contrast Memo KO MEFs migrated approximately 50% less (Figure 1C).

**Figure 2:**
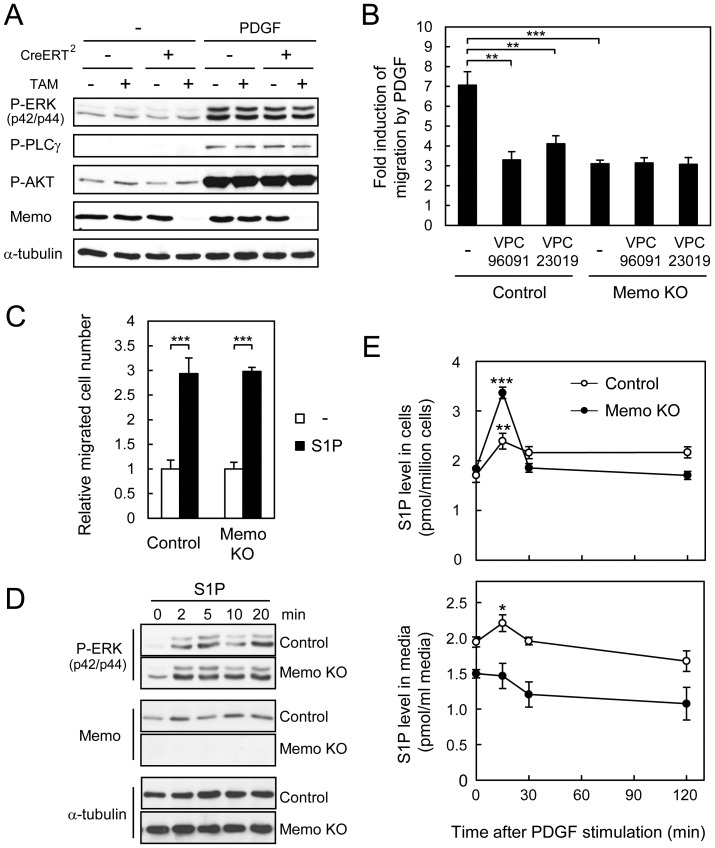
Memo KO MEFs have defects in cell-autonomous S1PR signaling induced by PDGF. **A**, MEFs with the indicated treatments were starved overnight and stimulated with PDGF (20 ng/ml) for 5 min. Western analyses were performed with the indicated antibodies. **B**, Transwell cell migration of control and Memo KO MEFs was induced by PDGF (5 ng/ml) in serum-free media with or without the SphK1-specific inhibitor, VPC96091 (1 μM) or the S1PR1/S1PR3-specific antagonist, VPC23019 (1 μM). **C**, Transwell cell migration of control and Memo KO MEFs was induced by S1P (5 nM) in serum-free media. In B and C, data were normalized to the average value for basal migration in serum-free media and are presented as means ± S.D. of three individual wells. Similar results were obtained in three independent experiments. **D**, Time course of ERK activation after S1P treatment of control and Memo KO MEFs. Control and Memo KO MEFs were starved overnight and stimulated with 1 μM S1P for the indicated time. Western analyses were performed with the indicated antibodies. **E**, S1P levels in control and Memo KO MEFs (upper panel) and their culture media (lower panel) after PDGF-stimulation. Starved cultures were treated with PDGF (50 ng/ml) and at the indicated time points, culture media and cells were harvested and analyzed for S1P levels by LC-ECI-MS/MS. Data are presented as means ± S.D. of three individual plates. In E, *p* values were calculated with respect to levels at the 0 time point. *, *p*<0.05; **, *p*<0.01; ***, *p*<0.001

The response of fibroblasts to PDGF is well characterized and PDGFR activation is known to stimulate multiple signaling pathways[28]. An examination of ERK, AKT, and PLCγ activation in control and Memo KO MEFs revealed no differences between the two cell lines (Figure 2A), suggesting that Memo does not influence activation of these pathways. PDGF also stimulates SphK1 and S1P production, and S1PR1 has been shown to be required for PDGF-induced migration[11], [12], [29]. Thus, we examined the role of cell-autonomous S1PR signaling in the MEFs by treating cells with PDGF in the presence of VPC96091, a SphK1 inhibitor[30], or with VPC23019, an S1PR1/3 antagonist. Both inhibitors significantly blocked PDGF-induced migration of control MEFs (Figure 2B), showing that this pathway is required. As shown in Figure 1C, Memo KO cells migrate less. Interestingly, neither of the inhibitors influenced PDGF-induced migration of the Memo KO MEFs (Figure 2B), suggesting that cell-autonomous signaling of the SphK1/S1PR1 axis is not active in these cells.

Next we analyzed the cells for changes that could explain the altered cell-autonomous signaling. The migratory response of control and Memo KO MEFs to exogenous S1P was the same (Figure 2C) and S1P-induced ERK activation was also similar in both cells lines (Figure 2D). There were no differences in the expression level of *Sphk2*, *S1pr1* and *S1pr3* in Memo control and KO MEFs (Figure S1A). Memo KO MEFs do, however, have significantly higher levels of *Sphk1* (Figure S1A) and SphK1 kinase activity (Figure S1B) compared to control MEFs. Thus, these data suggest SphK1 expression and activity, as well as S1PR signaling are intact in Memo KO MEFs. Increased expression of SphK1 might reflect the action of the Memo KO cells to compensate for the defects in cell-autonomous S1PR signaling. Finally, we analyzed S1P production dynamics by measuring intracellular and extracellular S1P levels in response to PDGF stimulation using a mass spectrometric approach. Intracellular S1P levels significantly increased within 15 min of PDGF treatment in both control and Memo KO MEFs (Figure 2E upper panel), showing that the pathway is intact. Indeed in the Memo KO MEFs S1P induction was higher than in control cells, likely reflecting elevated SphK1 kinase activity in these cells (Figure S1B). Extracellular S1P was detectable in the media of control and Memo KO MEFs in the absence of PDGF treatment (Figure 2E, lower panel) and in control cultures S1P levels were significantly increased after 15 min of PDGF treatment (Figure 2E, lower panel). In striking contrast there was no increase in extracellular S1P in the media of Memo KO MEFs (Figure 2E, lower panel), despite the robust induction of intracellular S1P. These results suggest that in the absence of Memo, cells fail to export S1P in response to PDGFR activation. Taken together the data suggest that the decreased migration observed in the Memo KO MEFs might be due to a defect in export of S1P or in the availability of S1P for cell-autonomous signaling.

### Memo knockdown endothelial cells have a defect in cell-autonomous S1PR signaling

**Figure 3:**
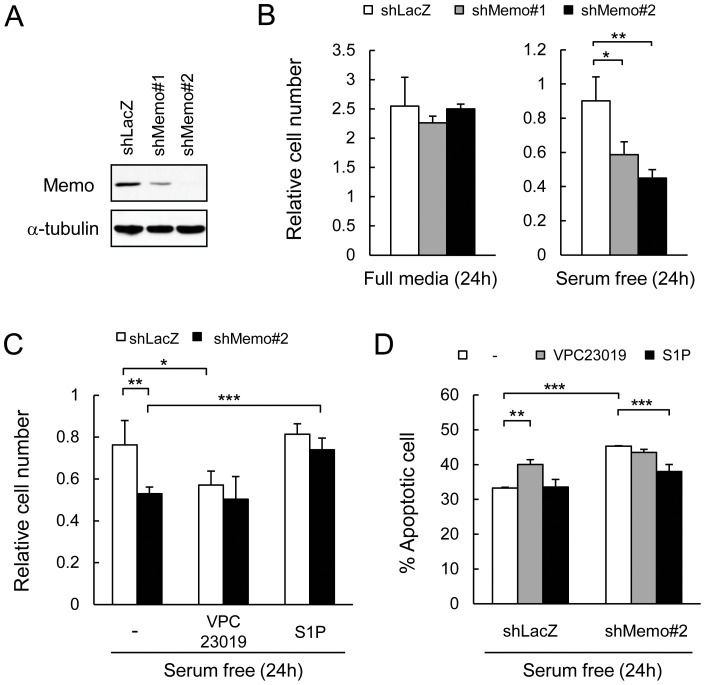
Defects in S1P signaling in Memo knock-down endothelial cells. **A**, HUVECs were infected with retroviruses expressing control shRNA (shLacZ) and two different shRNAs targeting Memo (shMemo#1 and #2). Western analyses were performed for Memo levels in the indicated cells. **B**, Control and Memo knock-down (KD) HUVECs were cultured in full media for 2 days and then cultured for another 24 hours (h) in full media (left panel) or serum-free media (right panel). Cells were counted and normalized to the average value at 2 days, which was set as 1. Data are presented as means ± S.D. of three individual plates. **C**, Effects of VPC23019 and S1P on survival of control and Memo KD HUVECs cultured in serum-free media. Cells were counted after 24 h in serum-free media with or without 1 μM VPC23019 or 1 μM S1P and normalized to the average value before switching to serum-free media, which was set as 1. Data are presented as means ± S.D. of four individual plates. **D**, Ratio of apoptotic cells/total cells after culturing 24 h in serum-free media with or without 1 μM VPC23019 or 1 μM S1P. Data are presented as means ± S.D. of four individual plates. *, *p*<0.05; **, *p*<0.01; ***, *p*<0.001

S1P is known to be important for endothelial proliferation, migration, angiogenesis and survival[18], which prompted us to examine Memo's role in endothelial cells. For this, Memo knock-down (KD) human umbilical vein endothelial cells (HUVECs) were generated using lentiviral vectors expressing 2 different shRNAs targeting Memo (shMemo#1 and shMemo#2). Both vectors decreased Memo levels, with vector #2 having the strongest effect (Figure 3A).

First, we examined migration of control and Memo KD HUVECs in response to VEGF. Both control and Memo KD cells robustly responded to VEGF (Figure S2A). We also observed no differences in VEGFR2 phosphorylation or ERK activation (Figure S2B) in response to VEGF when comparing control and Memo KD cells. When cultured in full media containing serum and growth factors, proliferation of control shLacZ cells and both Memo KD cell lines was the same (Figure 3B, left panel). When cells were cultured in serum-free media for 24 h, however, there were significantly fewer live cells in Memo KD cultures (Figure 3B, right panel). Serum is a major source of S1P, suggesting that these results might reflect a problem with cell-autonomous S1PR signaling in the absence of Memo. To examine this result further, cultures were exposed to serum-free media in the presence or absence of the S1PR1/3 antagonist, VPC23019. The number of control cells was significantly lower when S1PR1/3 activity was blocked (Figure 3C), pointing to a role for cell-autonomous S1PR signaling for proliferation or survival of HUVECs in conditions of low serum. In contrast, while there were fewer cells in the Memo KD cultures (as in Figure 3B), the addition of the S1PR1/3 antagonist did not cause a further decrease in cell number (Figure 3C).

The preceding results suggest that cell-autonomous S1PR1/3 activity is missing in the absence of Memo. To test this hypothesis, we examined the effect of exogenous S1P addition in serum-free conditions. There was no increase in cell number in the control cultures, while the number of Memo KD cells was significantly increased, up to the level of control, when S1P was added (Figure 3C, S1P bars). We also performed YO-PRO assays to check if apoptosis was responsible for the lower cell number. After 24 h of serum-free conditions, approximately 30% of control shLacZ cells were apoptotic, while there were significantly more apoptotic cells (45%) in the Memo KD cultures (Figure 3D, white bars). VPC23019 treatment significantly increased cell death, to 40% in control cultures, but had no effect on Memo KD cultures (Figure 3D, gray bars). The addition of S1P to control shLacZ cells had no effect on apoptotic-cell number, while S1P significantly increased survival of Memo KD cultures (8% decrease in apoptotic cells) (Figure 3D, black bars). These results clearly show that in serum-limiting conditions HUVECs rely at least in part on endogenous S1P and S1PR1/3 for their survival. This pathway is lacking in Memo KD cells rendering them more sensitive to serum withdrawal.

**Figure 4:**
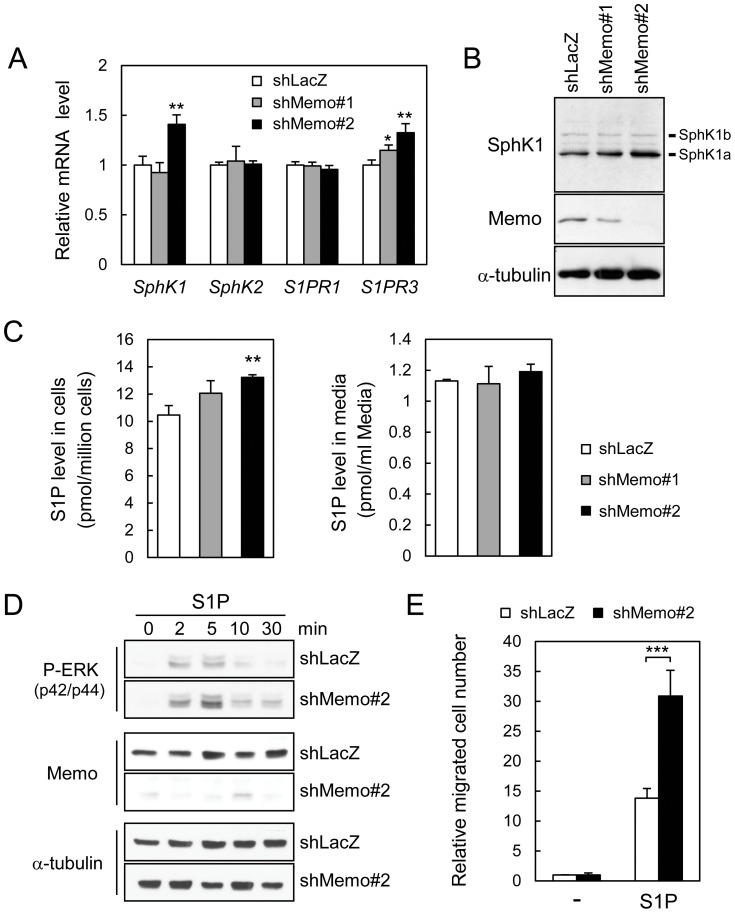
Analysis of S1P pathway and signaling in Memo knock-down endothelial cells. **A**, qPCR analysis for expression of *SphK1*, *SphK2*, *S1PR1* and *S1PR3* mRNA in HUVECs. Data were normalized to the average value for the control and are presented as means ± S.D. of three RNA samples extracted from three plates. **B**, Western analyses were performed for SphK1 and Memo levels in the indicated cells; the two splicing variant of SphK1 (a and b) are indicated. **C**, S1P levels in control and Memo KD HUVECs (left panel) and their culture media (right panel). After culturing 6 h in serum-free media, culture media and cells were harvested and analyzed for the levels of S1P by LC-ECI-MS/MS. Data are presented as means ± S.D. of three individual plates. **D**, Time course of ERK activation after S1P treatment of control and Memo KD HUVECs. HUVECs were starved for 6 h and stimulated with 1 μM S1P for the indicated time. Cell lysates were prepared and western analysis was performed with the indicated antibodies. **E**, Transwell cell migration of control and Memo KD HUVECs induced by S1P (100 nM) in serum-free media. The data were normalized to the average value for basal migration without S1P stimulation and are presented as means ± S.D. of five individual wells. *, *p*<0.05; **, *p*<0.01; ***, *p*<0.001

Next we examined if the Memo KD HUVECs have alterations in expression and/or activity of S1P signaling molecules. First, RNA levels of *SphK1*, *SphK2*, *S1PR1* and *S1PR3* were examined. Control and Memo KD HUVECs have similar levels of *SphK2* and *S1PR1*, however, the Memo KD cells show a significant increase in *SphK1* and *S1PR3* levels (Figure 4A). A western analysis with a SphK1 specific antiserum[31] also showed higher SphK1 protein levels, particularly in shMemo#2 KD cells (Figure 4B). To indirectly measure SphK activity, the level of S1P was determined using a mass spectrometric approach. In shMemo#2 KD cells, there were significantly higher intracellular S1P levels, in comparison to the other cells (Figure 4C, left panel), potentially due to higher SphK1 levels (Figure 4A–B). There were no significant differences in exogenous S1P levels in the three cell lines (Figure 4C, right panel). Next, we measured ERK signaling and migration of the HUVECs in response to exogenous S1P treatment. There was a rapid increase in ERK activity in response to S1P, with the shMemo#2 KD cells showing a more robust and prolonged increase in P-ERK levels compared to controls (Figure 4D). S1P also stimulated migration, showing a significantly stronger effect on the Memo KD HUVECs (Figure 4E). Thus, exogenous S1P signaling is clearly intact in control and Memo KD HUVECs. Indeed, the robust ERK activation and migration in the Memo KD HUVECs might be due to the higher levels of *S1PR3* in these cells (Figure 4A).

Taken together, these data indicate that the survival phenotypes in Memo KD HUVECs are not due to the defects in intracellular S1P production or S1PR signaling stimulated exogenously, but are likely to be caused by a defect in cell-autonomous S1PR activation. In summary, these results suggest that HUVECs require cell-autonomous S1PR signaling for survival in serum-limiting conditions and that Memo has a direct or indirect role in this pathway.

### Deletion of *Memo* causes vascular defects in developing embryos

**Figure 5:**
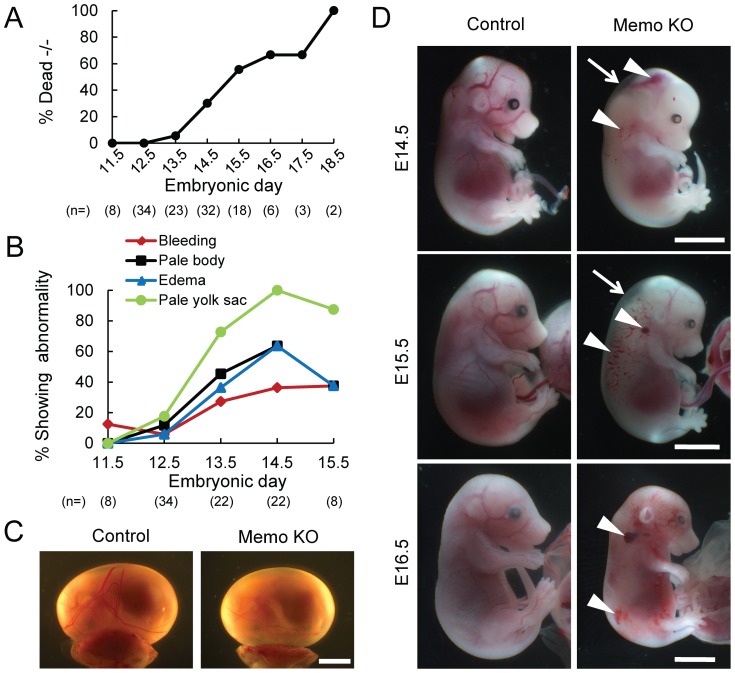
The phenotypes of control and Memo KO embryos. *Memo* +/− males and females were mated and the phenotypes of the *Memo* −/− (Memo KO) embryos were analyzed. **A**, Percentage of Memo KO embryos found dead with respect to total number of Memo KO embryos. **B**, percentage of living Memo KO embryos showing the indicated abnormality with respect to total number of living Memo KO embryos. In **A** and **B**, total sample numbers (n) for each stage are indicated at the bottom of each panel. **C**, Whole-mount view of control and Memo KO yolk sacs at E14.5. Scale bar, 4 mm. **D**, Whole-mount view of control and Memo KO embryos from E14.5 to E16.5. The sites of edema and bleeding frequently observed in Memo KO embryos are indicated as arrows and arrowheads, respectively. The Memo KO embryos shown in C and D were alive, except for E16.5. Scale bars, 4 mm. In all images, Memo KO is presented with the littermate control.

Memo is expressed during development and is evident throughout the embryos (Figure S3A–B) and in most organs from adult mice[4]. In order to gain insight into the physiological function of Memo, we generated *Memo* knock-out mouse strains. *Memo* +/− mouse strains were generated by conventional technology (Figure S4A–C). *Memo* +/− mice were healthy and fertile; however, no living *Memo* −/− pups were born from *Memo* +/− intercrosses, indicating embryonic lethality. To determine the timing and the cause of lethality, we analyzed embryos from *Memo* +/− intercrosses. As summarized in Table S2 and Figure 5A, *Memo* −/− embryos died starting from embryonic day 13.5 (E13.5); no live *Memo* −/− embryos were found at E18.5 (Figure 5A). Starting at E13.5 (Figure 5B) *Memo* −/− (Memo KO) embryos could be distinguished from controls (*Memo* +/+ and *Memo* +/−) by four characteristics (summarized in Figure 5B); pale yolk sacs (Figure 5C); pale embryos (Figure 5D); subcutaneous edema (Figure 5D arrows); and bleeding, especially in the head and neck regions (Figure 5D arrowheads). In addition, some of the dead embryos were filled with blood (Figure 5D, E16.5).

**Figure 6:**
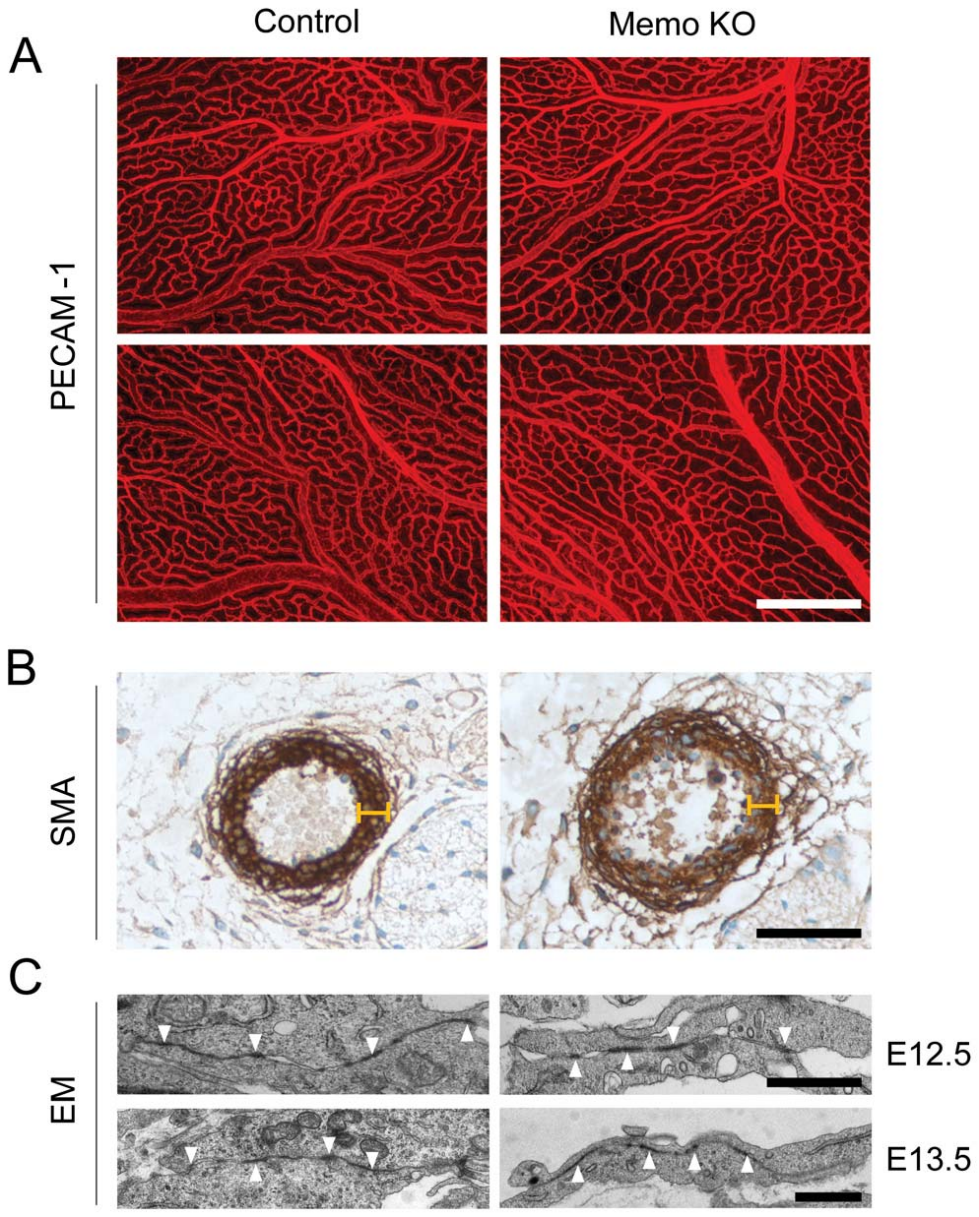
Histological analyses of the vasculature in control and Memo KO embryos. **A**, Whole-mount immunostaining with anti-CD31 (PECAM-1) antibody on control and Memo KO yolk sacs at E13.5. Scale bar, 500 μm. Representatives images of two different regions are shown. **B**, Immunostaining with anti-smooth muscle actin (SMA) antibody on dorsal aorta of control and Memo KO embryos at E14.5. Note that the dorsal aorta of the Memo KO embryo was covered by a layer of smooth muscle cells of the same width as control (indicated with the markers of the same scale). Scale bar, 50 μm. **C**, Electron microscopy micrographs (EM) of tight junctions (indicated as arrowheads) formed in limb bud capillary vessels in control and Memo KO embryos at E12.5 (upper panel) and E13.5 (lower panel). Scale bar, 1 μm. Only Memo KO embryos with obvious abnormalities (pale yolk sac and bleeding) were used for the analyses. In all images, Memo KO is presented with the littermate control.

Immunohistochemical studies revealed that Memo KO yolk sacs were paler than controls, but had an apparently normal vasculature organization with normal branched vessels (Figure 6A). Embryos with bleeding phenotypes during late embryogenesis often have defects in smooth muscle coverage of the vessels[32], [33]. We examined the dorsal aorta, a large vessel, and observed slight dilation, however, the vessels were covered by an intact layer of smooth muscle cells (Figure 6B). These data indicate that typical steps for vasculogenesis, angiogenesis and vascular maturation appear to be normal in Memo KO embryos. Furthermore, EM analysis showed no differences in the appearance of tight junctions formed in capillary blood vessels between control and Memo KO embryos (Figure 6C). Thus, although vasculature defects are likely to contribute to the lethality observed in Memo KO embryos, we could not identify an obvious reason for the bleeding phenotype.

### Memo acts within endothelial cells to regulate vascular development

**Figure 7:**
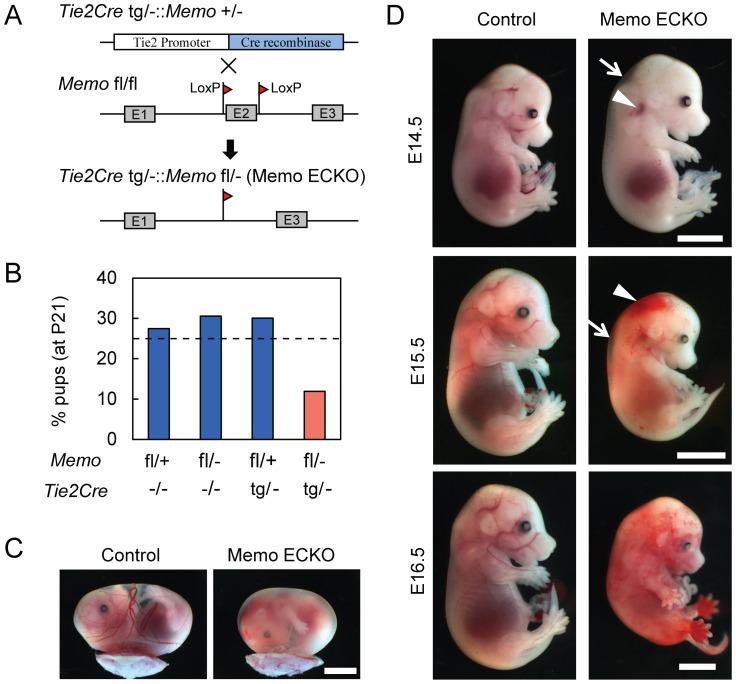
The phenotypes of endothelial cell-specific Memo KO embryos. **A**, Schematic diagram for generating mice specifically lacking Memo in endothelial cells. *Tie2Cre* tg/−::*Memo* +/− males were mated with *Memo* floxed/floxed (fl/fl) females and the resulting *Tie2Cre* tg/−::*Memo* fl/− pups were analyzed as Memo endothelial cell-specific knock-out (ECKO) mice. **B**, Ratio of the genotypes of pups (n = 193) at postnatal day 21 (P21). The expected ratio of each genotype (25%) is indicated as a dashed line. **C**, Whole-mount view of control and Memo ECKO yolk sacs at E15.5. Scale bar, 4 mm. **D**, Whole-mount view of control and Memo ECKO embryos from E14.5 to E16.5. The sites of edema and bleeding are indicated as arrows and arrowheads, respectively. Scale bars, 4 mm. All Memo ECKO embryos shown in C and D were alive except for E16.5. In all images, Memo KO is presented with the littermate control.

To specify a cell type that might be responsible for the phenotypes, we generated Memo endothelial cell-specific KO (ECKO) mice by crossing *Memo* fl/fl mice with *Tie2Cre* transgenics[27]. *Tie2Cre* tg/−::*Memo* +/− males were crossed with *Memo* fl/fl females to generate Memo ECKO mice (Figure 7A). Genomic PCR analysis of *Memo* in the offspring revealed that Tie2Cre was active since crosses of *Tie2Cre* tg/−::*Memo* +/− with *Memo* fl/+ displayed a band corresponding to the deleted allele (Figures S5A, lane 3). All possible genotypes were identified in pups at postnatal day 21 (P21) (Figure S5A and Figure 7B), however, the ratio of *Tie2Cre* tg/−::*Memo* fl/− (Memo ECKO) was only 10%, i.e., lower than the expected 25%, suggesting that >50% of the Memo ECKO null embryos died (Figure 7B). Surviving Memo ECKO pups developed into adults; the only obvious abnormality was their lower weight compared to control mice (Figure S5B).

A close examination of the embryos resulting from the *Tie2Cre* tg/−::*Memo* +/− x *Memo* fl/fl crosses revealed that some Memo ECKO embryos had the same features as *Memo* −/− embryos, including pale yolk sacs (Figure 7C), subcutaneous edema and significant bleeding (Figure 7D). Moreover, the onset of these features at E13.5 is similar to that seen in the *Memo* −/− embryos. These observations indicate that Memo plays an important role within endothelial cells to regulate vascular development. The different penetrance of lethality observed in the Memo ECKO model suggests that there might be additional defects in the *Memo* −/− embryos, a result which is not unexpected considering that Memo is widely expressed.

### Memo knockdown endothelial cells have a defect in S1P-mediated junctional localization of VE-cadherin and exhibit hypersprouting

The observed phenotypes and the timing of their onset in Memo KO and ECKO embryos have some of the features described for embryos lacking proteins in the S1P signaling network[19]–[21]. KO of S1PR1 in embryos as well as in endothelial cells causes rapid embryonic death between E12.5 and E14.5, with prominent bleeding and defects in aortic vascular smooth muscle cell coverage[20], [34]. Neither the embryos with full Memo KO nor the ECKO embryos have such a severe phenotype. It is interesting, however, that starting at E13.5 S1PR2/S1PR3 double KO mice show partial embryonic lethality and hemorrhaging, but have no obvious defects in smooth muscle cell recruitment around large vessels[21]; characteristics that are quite similar to those observed in embryos from Memo KO and ECKO.

**Figure 8:**
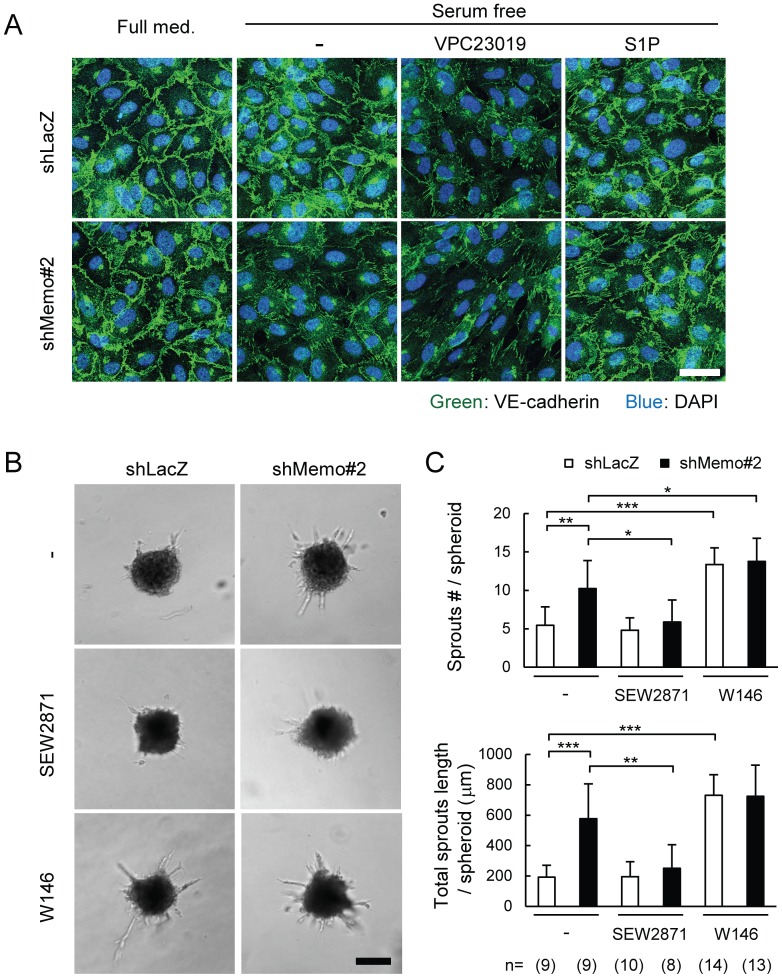
Defects in stabilization of VE-cadherin-mediated cellular junctions in Memo knock-down endothelial cells. **A**, Effects of S1P or S1P receptor blocker on junctional localization of VE-cadherin in control and Memo KD HUVECs. Monolayers of HUVECs were cultured for 6 h in full media or serum-free media with or without VPC23019 (1 μM) or S1P (1 μM), then fixed and stained for VE-cadherin. Scale bar, 40 μm. **B–C**, Sprout formation from multicellular spheroids generated from control and Memo KD HUVECs. Sprouting was compared in the absence and presence of the S1PR1-specific agonist, SEW2871 (100 nM) or the S1PR1-specific antagonist, W146 (10 μM). Representative images (B) and quantified results (C) are shown. The numbers of multicellular spheroids used for each condition are indicated at the bottom of (C). Scale bar in (B), 100 μm. Data in (C) are presented as means ± S.D. of the scores for each multicellular spheroid. *, *p*<0.05; **, *p*<0.01; ***, *p*<0.001

S1PR1 was recently shown to be important for stabilization of VE-cadherin at endothelial junctions[16], [17]. In the final experiments we examined the impact of Memo loss on endothelial junctions in the HUVEC model. Immunofluorescent (IF) staining revealed that control and Memo KD cells show similar levels of VE-cadherin positive junctions when cultured in full medium (Figure 8A), which is consistent with the *in vivo* EM results (Figure 6C). Notably, however, after culturing 6 h in serum-free medium there was a dramatic loss of VE-cadherin staining in the junctions of the Memo KD HUVECs, while VE-cadherin staining in control cultures was unaltered (Figure 8A, -). This is likely to be due to VE-cadherin relocalization and not to its loss since the level of VE-cadherin in control and Memo KD cells in serum-free medium is the same (Figure S6A). Addition of the S1PR1/3 antagonist VPC23019 reduced the signal of junctional VE-cadherin in control cultures, but had no obvious effect on Memo KD HUVECs (Figure 8A, VPC23019). Conversely, exogenous S1P rescued the phenotype of Memo KD cells, but had no impact on VE-cadherin staining in control cultures (Figure 8A, S1P). These results show that in control HUVECs cell-autonomous S1PR signaling is required for stabilizing junctional VE-cadherin in conditions with little or no exogenous S1P. Moreover, the Memo KD HUVECs have a defect in this pathway and this is rescued by exogenous S1P.

Hypersprouting of the vascular network has been observed when vascular junctions are destabilized due to defective S1PR1 signaling[16], [17]. To examine if Memo loss affects sprouting, we monitored sprout formation from HUVEC multicellular spheroids in S1P-reduced conditions. Spheroids of Memo KD HUVECs gave rise to sprouts significantly more frequently and the total sprout lengths were longer compared to control spheroids (Figure 8B–C). Importantly the addition of the S1PR1-specific agonist SEW2871[35] lowered the number and the length of the sprouts in Memo KD spheroids back to the levels measured in control spheroids (Figure 8B–C); control spheroids were not affected by the agonist (Figure 8B–C). In contrast to the effect with the agonist, the S1PR1-specific blocker W146[36] significantly stimulated the sprouting number and length of control HUVEC spheroids, while its effects on the Memo KD spheroids were minimal (Figure 8B–C). Moreover, sprouting of control HUVECs, but not the Memo KD spheroids, was also stimulated with the S1PR1/3 blocker VPC23019 (Figure S6B–C).

In summary, taken together these results suggest that there are defects in S1PR1 signaling in Memo's absence. Indeed, they suggest that Memo plays a role in stabilizing junctional VE-cadherin through the activation of S1P signaling in a cell-autonomous manner. In its absence, junctions are destabilized and increased sprouting of HUVEC multicellular spheroids was observed.

## Discussion

**Figure 9:**
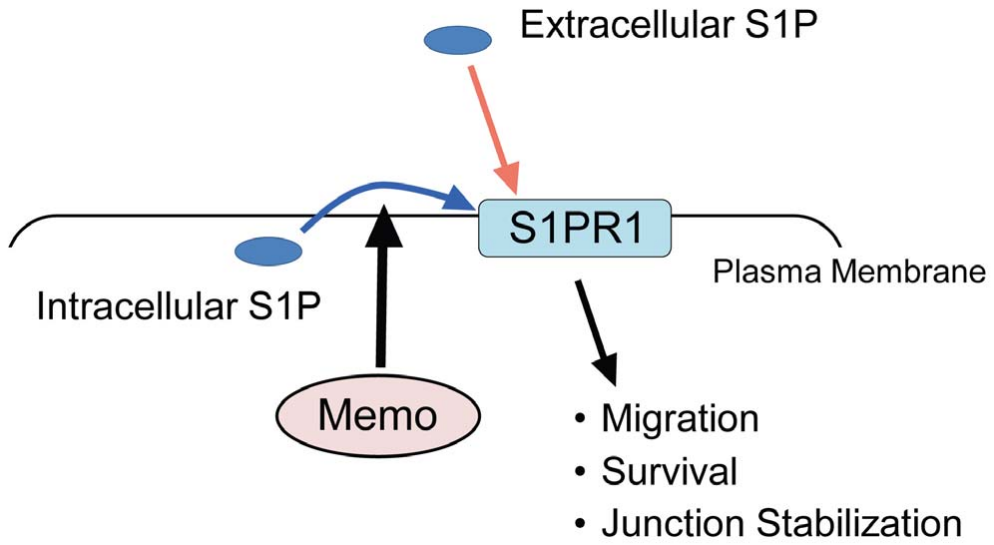
Memo has an important role in cell-autonomous S1PR signaling. S1PR1 can be activated by extracellular (red arrow) and intracellular (blue arrow) S1P. Our *in vitro* data demonstrate that Memo has an important role in the cell-autonomous pathway activating S1PR1 by intracellular S1P. In MEFs, absence of Memo results in a defect in cell-autonomous activation of S1PR1 during cell migration induced by PDGF. In HUVECs, knockdown of Memo results in defects in survival and junction stabilization, both of which were rescued by addition of exogenous S1P.

Our group identified Memo as an essential protein required for breast cancer cell motility in response to RTKs. Memo KD tumor cells showed decreased migration upon treatment with EGF, HRG or FGF[1], [2]. Mechanistically, we know that Memo is required for localization of RhoA and mDia1 to the cell cortex, which promotes microtubule (MT) outgrowth in cellular protrusions and migration[37]–[39]. In the work presented here we uncovered a novel role for Memo in S1P signaling. Our results from experiments carried out with Memo null MEFs and Memo KD HUVECs lead to the conclusion that Memo is required, either directly or indirectly, for cell-autonomous S1PR signaling (Figure 9). PDGF is well-known to require cell-autonomous signaling of the SphK1/S1PR1 axis to induce migration[11], [12], [29]; our data clearly show that this pathway is not active in cells lacking Memo. S1P is also known to be important for endothelial cell proliferation and survival[18]. We show that in conditions of serum-deprivation Memo KD HUVECs have decreased survival and loss of junctional stability; both phenotypes were rescued by exogenous S1P, also pointing to a problem with cell-autonomous S1PR signaling in Memo's absence. Considering the importance of S1P signaling in numerous pathophysiological processes[5]–[7], we think that the discovery of a novel player in this pathway is very relevant.

One important feature of Memo KO/KD cells is that they have no defect in the response to extracellular S1P or in the intracellular production of S1P. Their response to S1P is intact with respect to signaling pathway activation and migration. Furthermore, no defect is observed in their basal intracellular S1P level. In Memo KO MEFs, we have observed clear induction of intracellular S1P level upon stimulation with PDGF. These results rule out the possibility that Memo is required downstream of S1PRs or for the activation of SphKs. How might Memo control cell-autonomous S1PR signaling? The exact mechanism is still unknown, but based on our results we favor a model in which Memo might regulate access of intracellular S1P to the S1PR1 (Figure 9). In response to PDGF treatment, control MEFs, but not the Memo KO MEFs showed an increase in extracellular S1P levels. This suggests that Memo loss somehow impairs transport of S1P, which is produced and exported in a situation of acute RTK activation. However, in the case of HUVECs in non-stimulated conditions, no difference was detected in extracellular S1P levels between control and Memo KD cultures. Given that both cellular systems, the MEFs and the HUVECs, have defects in cell-autonomous S1PR signaling, the results suggest that S1P transport that is Memo-mediated, is not necessarily its release to the extracellular milieu. The crystal structure of S1PR1[40] provides an interesting explanation for such a process. The structural analysis suggests that access to the ligand-binding pocket of S1PR1 could be achieved from within the plasma membrane. This would allow intracellular S1P to bind the receptor during transport from the inner to the outer leaflet of the plasma membrane; extracellular release might not be necessary[40]. Thus, Memo might be somehow involved in local transporting events that allow efficient access of S1P to S1PR1. Further experimentation will be necessary to test this hypothesis.

It has been shown in different cell types that the ABC family of transporters is involved in S1P transport[9]; in endothelial cells the non ATP-dependent transporter Spns2 has been shown to be essential[41]. It is unlikely that Memo KO/KD impairs Spns2 since, as just mentioned, the levels of extracellular S1P are similar in control and Memo KD HUVECs. Moreover there is no major difference in S1P levels in Memo KO embryos; the lower levels observed at E12.5, were recovered by E13.5 (Figure S4D) and Memo ECKO null mice and control mice have similar levels of plasma S1P (Figure S5C). These results are in contrast to the Spns2 ECKO mice which show no lethal defect during embryogenesis, but do display decreased plasma S1P levels[41], [42]. We cannot rule out the possibility that Memo does have a role in regulating some of the other ABC transporters.

Another interesting possibility to discuss is that Memo has a role in coordinating events promoting S1P-S1PR1 binding by regulating localization of SphK1, S1P transporters, and S1PR1. It has been proposed that their localization is coordinated with actin dynamics through the function of an actin binding protein filamin A[15]. Memo has been shown to have a role in coordinating the dynamics of the microtubule and actin networks[37]. Although recombinant purified Memo and SphK1 do not directly interact (Figure S7B), we were able to pull-down complexes of Memo and SphK1 from lysates of HEK293 cells transfected with Memo and SphK1 expression vectors (Figure S7A). Thus, the observed defect in cell-autonomous S1PR signaling might reflect defects in the coordinated localization of SphK, S1P transporters and the receptors.

The second important result presented in this manuscript is the discovery that Memo is an essential gene for embryonic development; all *Memo* −/− embryos died between E13.5 and E18.5. Moreover, we show that Memo ECKO embryos have features similar to the full KO, including edema and significant bleeding. These observations indicate that Memo plays an important role within endothelial cells and that vasculature defects are likely to contribute to lethality, although an obvious reason for the bleeding phenotype was not uncovered. The observed phenotypes in Memo KO embryos and their timing of onset are similar to what has been reported for S1PR1 KO and S1PR2/S1PR3 double KO mice. S1PR1 KO embryos, however, have the most severe phenotype, with prominent bleeding and defects in aortic vascular smooth muscle cell coverage and rapid death. In contrast, S1PR2/S1PR3 double KO mice show partial embryonic lethality and hemorrhaging, with no obvious defects in aortic smooth muscle cell coverage[21], very likely reflecting compensation by S1PR1. It is tempting to speculate that *in vivo* Memo also has an important role in S1P signaling during embryonic development, however, we cannot link our *ex vivo* findings on Memo's involvement in cell-autonomous S1PR signaling with the embryonic phenotypes. Despite the fact that the biological significance of cell-autonomous S1PR signaling has been observed in many cellular studies[6], [10], including those shown here, the *in vivo* contribution of this pathway to normal physiology has not been conclusively shown. Erythrocytes and endothelial cells provide S1P in the blood so that S1PRs are readily activated by external sources [43], [44]. Finally, it is worth mentioning that no embryos survive beyond E18.5 in the absence of Memo, while Memo loss in endothelial cells is less severe, with ∼50% of the pups surviving. The embryonic lethal phenotype may reflect Memo's importance in other developmental processes. Memo has been found associated with signaling from ErbB2[1], FGFR[4], PDGFR (shown here) and IGF1R[45]. Each of these receptors have essential developmental roles, however, none of the phenotypes that we have found are similar to those described for these receptors. Thus, the lethal phenotype we describe here is likely to arise from a still to be identified role for Memo.

In conclusion, our data show that Memo has an important role in cell-autonomous S1PR signaling. Future studies will be aimed at further elucidation of the molecular basis of Memo's role in cell-autonomous S1PR signaling. S1P signaling is involved in different diseases like autoimmunity or cancer and is predicted to be an important therapeutic target. Indeed, several drugs targeting SphKs, S1PRs or S1P are now under clinical investigation [46]. Our discovery of a new player in this pathway suggests that in the future there might be additional options to target the S1PR pathway.

## Supporting Information

Figure S1 **A**, qPCR analysis for expression of *Sphk1*, *Sphk2*, *S1pr1* and *S1pr3* mRNA in control and Memo KO MEFs. Data were normalized to the average value for the control and are presented as means ± S.D. of three RNA samples extracted from three plates. **B**, SphK1 activity in control and Memo KO MEFs. Equal amounts of cytoplasmic lysates from control and Memo KO MEFs were analyzed for SphK1 activity. Data were normalized to the average value for the control, which is set as 1, and are presented as means ± S.D. of samples from three individual plates. *, *p*<0.05; **, *p*<0.01.
(TIF)

Figure S2 **A**, Transwell cell migration of control and Memo KD HUVECs induced by VEGF (20 ng/ml). The data were normalized to the average value for basal migration without VEGF stimulation and are presented as means ± S.D. of five individual wells. **B**, Time course of VEGFR2 and ERK activation after VEGF treatment of control and Memo KD HUVECs. Monolayers of HUVECs was starved for 6 h and stimulated with 100 ng/ml VEGF for the indicated time. Cell lysates were prepared and western analyses were performed with the indicated antibodies; α-tubulin is the loading control.
(TIF)

Figure S3 **A**, Expression of Memo in mouse embryos. Lysates were prepared from whole mouse embryos of the indicated stages and western analyses were performed for Memo levels. **B**, Detection of *Memo* mRNA in mouse embryos by *in situ* hybridization. Sagittal sections of mouse embryos of the indicated stages were hybridized with *Memo* antisense and sense (control) riboprobe.
(TIF)

Figure S4 **A**, Schematic diagram for generating Memo KO mice. Wild-type alleles in mouse ES cells were targeted with a targeting construct containing a floxed NEO cassette and exon 2 (E2) of the mouse *Memo* gene. An ES clone containing the targeted allele was selected and subsequently transfected with an expression vector for Cre recombinase to delete the NEO cassette and E2 of *Memo*. ES clones containing the deleted allele were selected and used for chimera production. A mouse line giving germ-line transmission with the ES clone was further bred as Memo +/−. PCR primer F and R were used for genotyping. **B**, Genomic PCR analysis for the *Memo* gene carried out on DNA extracted from embryos from Memo +/− intercrosses. The fragments amplified from the wild-type (wt) and deleted (del) allele of *Memo* are indicated. The three possible genotypes, i.e. +/+, +/− and −/− are represented. **C**, Expression of Memo protein in control and Memo KO embryos. Tissue extracts from whole embryos (E11.5) with the indicated genotypes were prepared and western analyses were performed for Memo. **D**, S1P levels in control and Memo KO mouse embryos. S1P was extracted from whole embryos of the indicated stages and analyzed by LC-ECI-MS/MS. Data are presented as means ± S.D. **, *p*<0.01.
(TIF)

Figure S5 **A**, Genomic PCR analysis of the *Memo* gene carried out on ear DNA of the pups at P21. The fragments amplified from the floxed (flox), wild-type (wt) and deleted (del) allele of the *Memo* gene are indicated. The four possible genotypes i.e. 1) *Memo* fl/+::*Tie2Cre* −/−, 2) *Memo* fl/−::*Tie2Cre* −/−, 3) *Memo* fl/+:: *Tie2Cre* tg/− and 4) *Memo* fl/−:: *Tie2Cre* tg/− are represented. **B**, Body weight of control and *Memo* ECKO mice. Data are presented as means ± S.E. **C**, S1P level in control and Memo KO mouse plasma. S1P was extracted from plasma of adult mice (12-weeks old) and analyzed by LC-ECI-MS/MS. Data are presented as means ± S.D. *, *p*<0.05; **, *p*<0.01; ***, *p*<0.001.
(TIF)

Figure S6 **A**, Expression of VE-cadherin in control and Memo KD HUVECs after starvation. Monolayers of HUVECs were cultured in serum-free media for 6 h. Cell lysates were prepared and western analyses were performed with the indicated antibodies. **B–C**, Sprout formation from multicellular spheroids generated from control and Memo KD HUVECs. Sprouting was compared in the absence and presence of VPC23019 (10 μM). Representative images (B) and quantified results (C) are shown. A part of the data shown in Figure 8B and 8C is shown again in order to compare the effect of non-treated shLacZ and shMemo#2 to VPC23019. The numbers of multicellular spheroids used for each condition are indicated at the bottom of (C). Scale bar in (B), 100 μm. Data in (C) are presented as means ± S.D. of the scores for each multicellular spheroid. *, *p*<0.05; **, *p*<0.01; ***, *p*<0.001.
(TIF)

Figure S7 **A**, Complex formation between Memo and SphK1 in HEK293T cells. Cells were transiently transfected with vectors expressing myc-Memo and/or V5-SphK1. After 48 h, whole-cell lysates (WCL) were prepared and subjected to immunoprecipitation (IP) using either an anti-V5 or anti-myc antibody. Western analyses were performed using the indicated antibodies. **B**, Recombinant Memo and SphK1 do not directly interact. Recombinant Memo (2 μg) or myc-Memo (2 μg) was incubated with, or without, recombinant His-SphK1 (2 μg) and the protein mixtures were subjected to pull-down assays using Ni-NTA agarose. After washing, the bound proteins were eluted from the agarose and western analyses were performed using the indicated antibodies. 50 ng of each protein was loaded as a control.
(TIF)

Table S1 **Primer pairs used for qPCR.**
(PDF)

Table S2 **Analysis of embryos from Memo** +/− **intercrosses.** *Memo* +/− males and females were mated and the genotype of the offspring at the indicated ages was analyzed. The number of embryos with the indicated genotype are listed; the numbers of dead embryos, as judged by the absence of a heartbeat is in the (). ^*^ % live *Memo* −/−/total live embryos.
(PDF)
