# Correction: Memo Has a Novel Role in S1P Signaling and Crucial for Vascular Development

**DOI:** 10.1371/journal.pone.0101351

**Published:** 2014-06-26

**Authors:** The PLOS ONE Staff

The word “is” is missing in the article title. The correct title is: Memo Has a Novel Role in S1P Signaling and is Crucial for Vascular Development. The correct citation is: Kondo S, Bottos A, Allegood JC, Masson R, Maurer FG, et al. (2014) Memo Has a Novel Role in S1P Signaling and is Crucial for Vascular Development. PLoS ONE 9(4): e94114. doi:10.1371/journal.pone.0094114
