# Supplementary figures and images for: Memo Has a Novel Role in S1P Signaling and Crucial for Vascular Development
Source: PLoS One. 2014 Apr 8;9(4):e94114. doi: 10.1371/journal.pone.0094114 (PMC3979765; doi:10.1371/journal.pone.0094114)

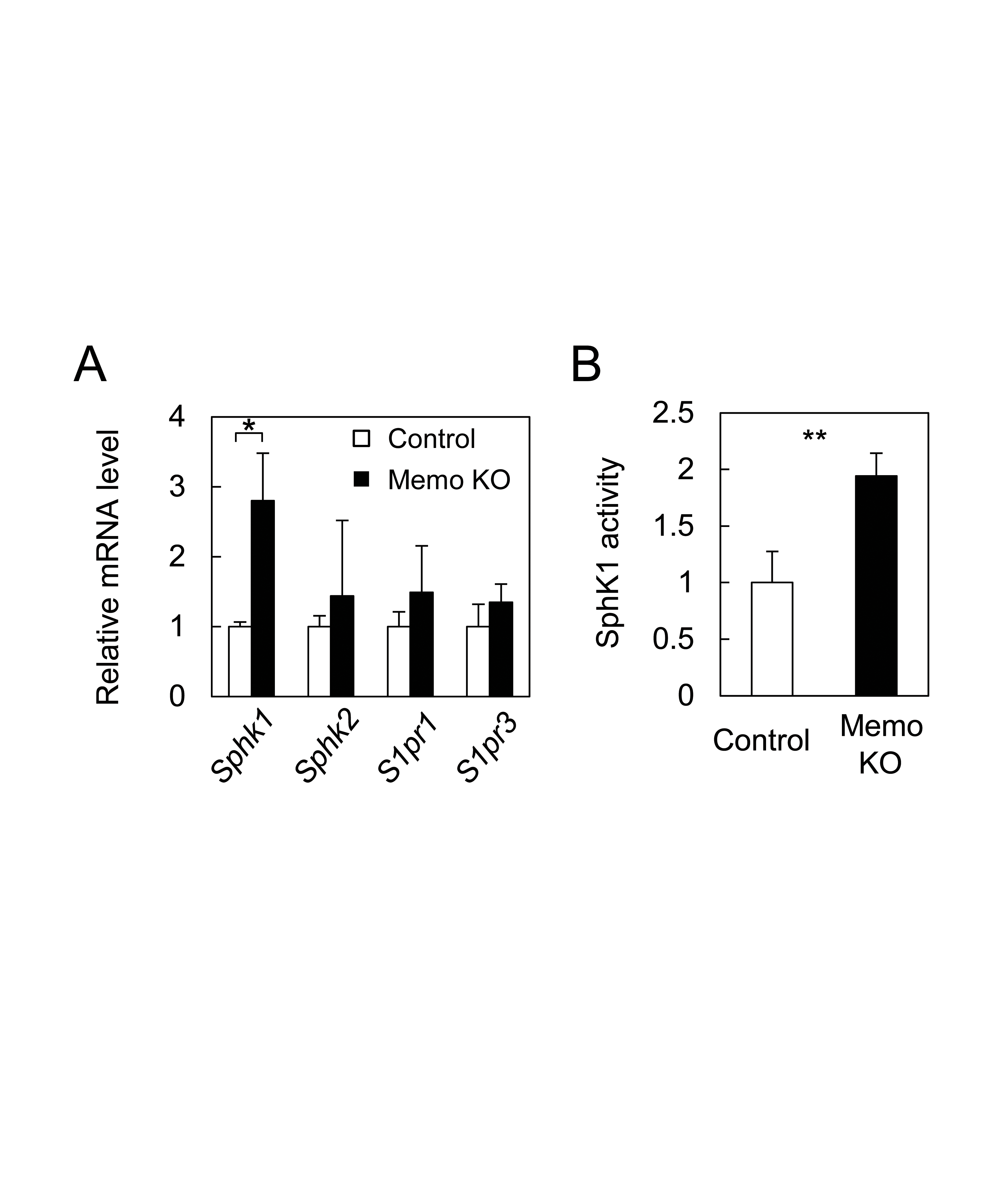

Supplement: Figure S1 — A, qPCR analysis for expression of Sphk1, Sphk2, S1pr1 and S1pr3 mRNA in control and Memo KO MEFs. Data were normalized to the average value for the control and are presented as means ± S.D. of three RNA samples extracted from three plates. B, SphK1 activity in control and Memo KO MEFs. Equal amounts of cytoplasmic lysates from control and Memo KO MEFs were analyzed for SphK1 activity. Data were normalized to the average value for the control, which is set as 1, and are presented as means ± S.D. of samples from three individual plates. *, p<0.05; **, p<0.01. (TIF) [file pone.0094114.s001.tif]

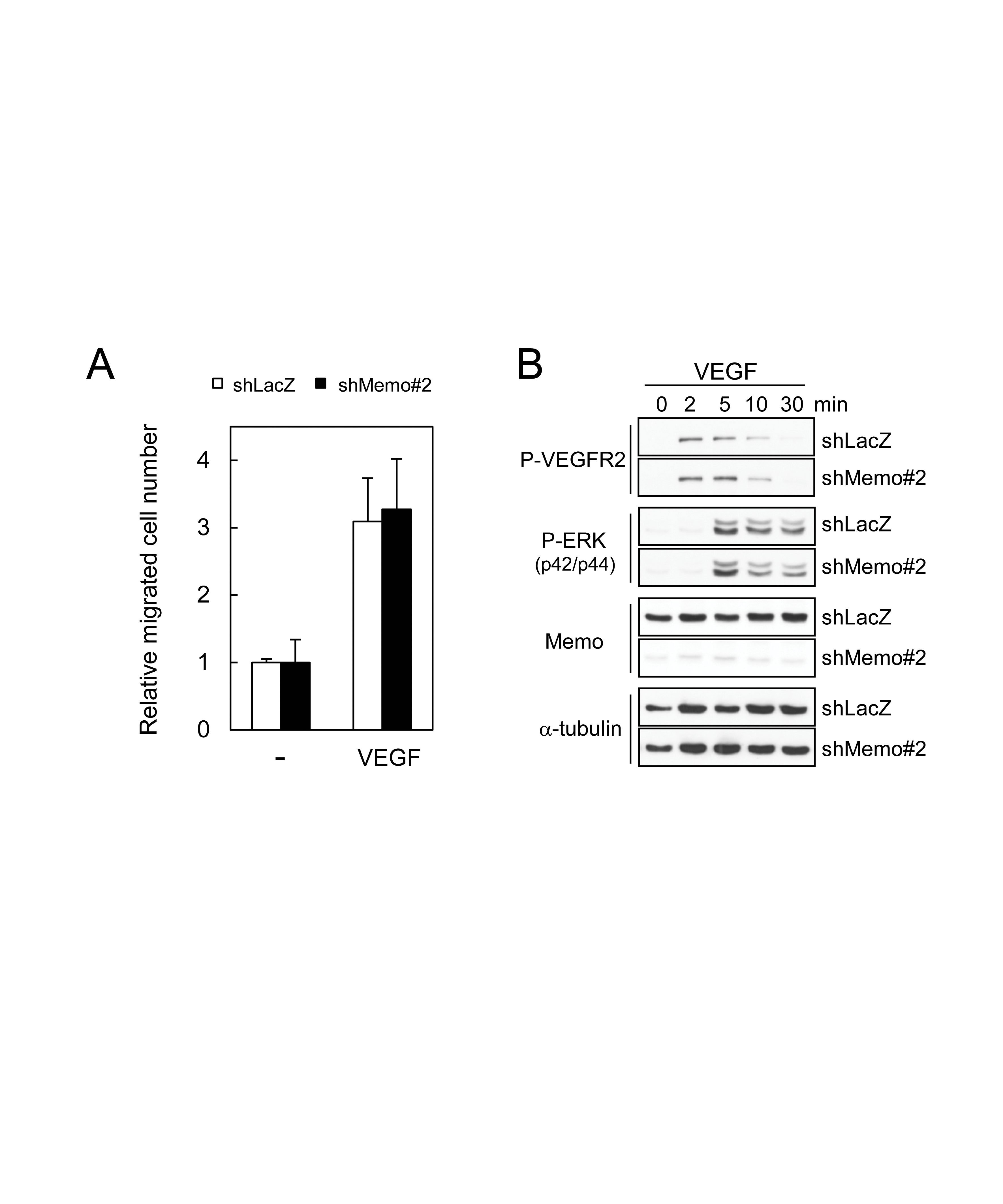

Supplement: Figure S2 — A, Transwell cell migration of control and Memo KD HUVECs induced by VEGF (20 ng/ml). The data were normalized to the average value for basal migration without VEGF stimulation and are presented as means ± S.D. of five individual wells. B, Time course of VEGFR2 and ERK activation after VEGF treatment of control and Memo KD HUVECs. Monolayers of HUVECs was starved for 6 h and stimulated with 100 ng/ml VEGF for the indicated time. Cell lysates were prepared and western analyses were performed with the indicated antibodies; α-tubulin is the loading control. (TIF) [file pone.0094114.s002.tif]

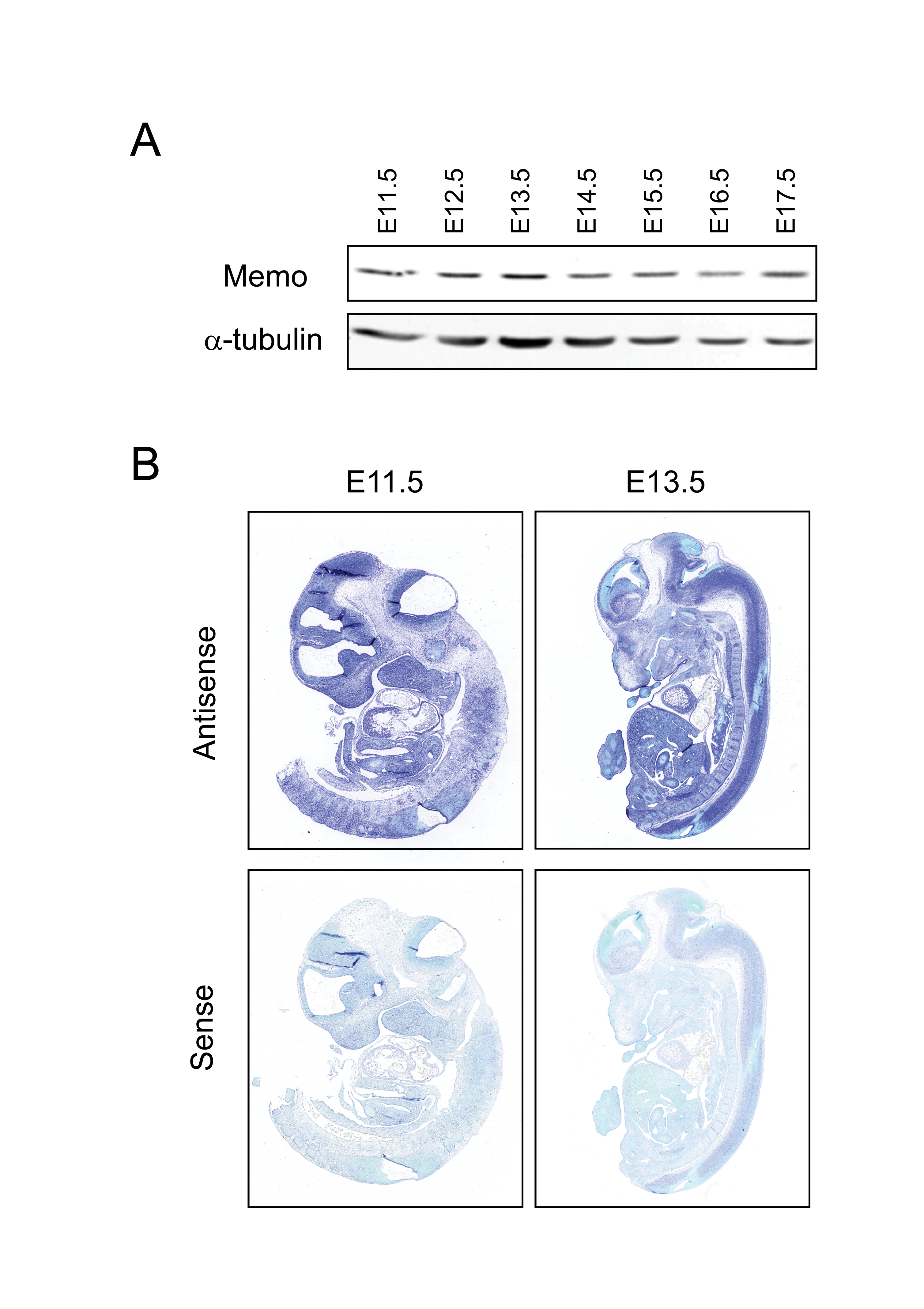

Supplement: Figure S3 — A, Expression of Memo in mouse embryos. Lysates were prepared from whole mouse embryos of the indicated stages and western analyses were performed for Memo levels. B, Detection of Memo mRNA in mouse embryos by in situ hybridization. Sagittal sections of mouse embryos of the indicated stages were hybridized with Memo antisense and sense (control) riboprobe. (TIF) [file pone.0094114.s003.tif]

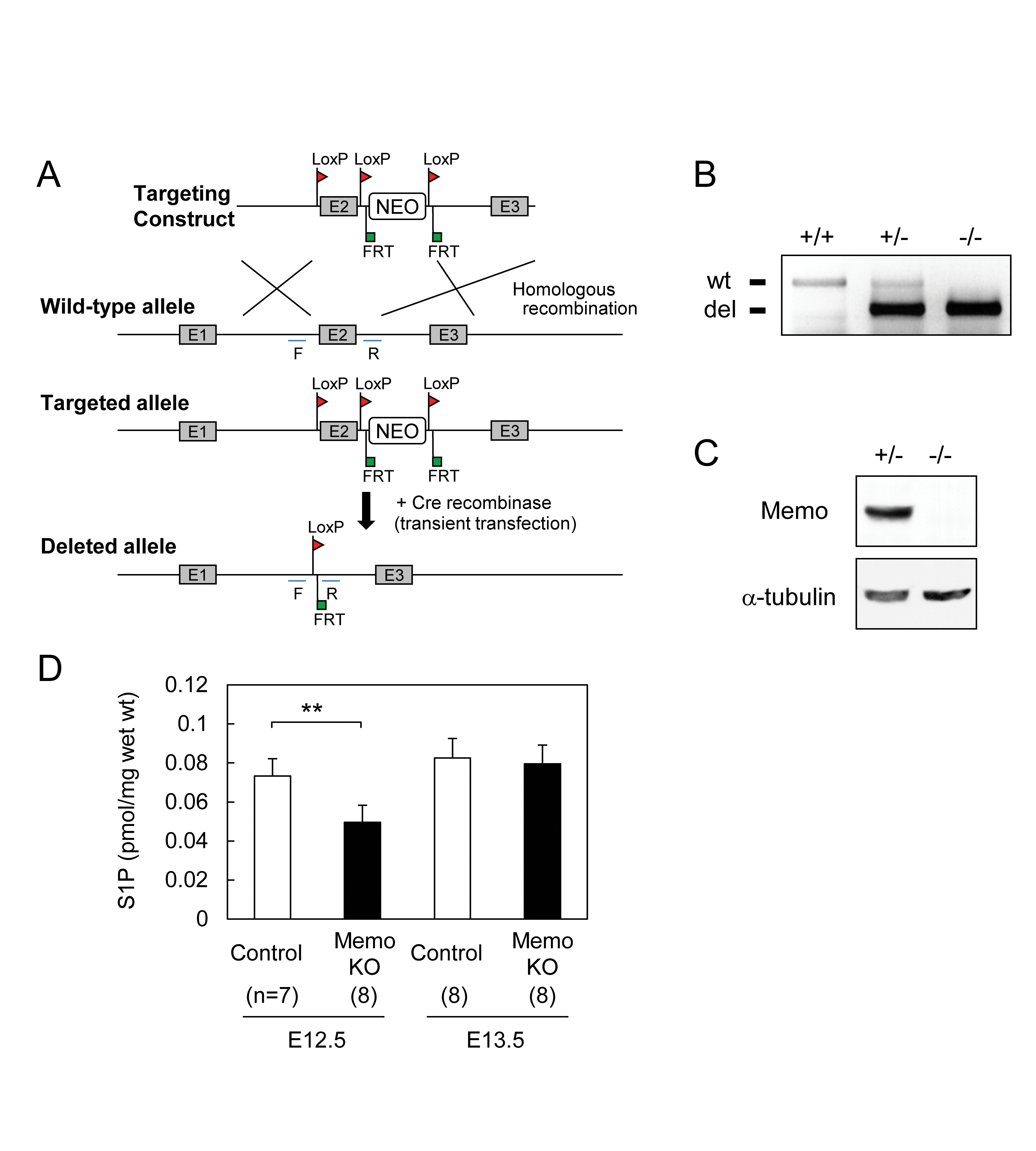

Supplement: Figure S4 — A, Schematic diagram for generating Memo KO mice. Wild-type alleles in mouse ES cells were targeted with a targeting construct containing a floxed NEO cassette and exon 2 (E2) of the mouse Memo gene. An ES clone containing the targeted allele was selected and subsequently transfected with an expression vector for Cre recombinase to delete the NEO cassette and E2 of Memo. ES clones containing the deleted allele were selected and used for chimera production. A mouse line giving germ-line transmission with the ES clone was further bred as Memo +/−. PCR primer F and R were used for genotyping. B, Genomic PCR analysis for the Memo gene carried out on DNA extracted from embryos from Memo +/− intercrosses. The fragments amplified from the wild-type (wt) and deleted (del) allele of Memo are indicated. The three possible genotypes, i.e. +/+, +/− and −/− are represented. C, Expression of Memo protein in control and Memo KO embryos. Tissue extracts from whole embryos (E11.5) with the indicated genotypes were prepared and western analyses were performed for Memo. D, S1P levels in control and Memo KO mouse embryos. S1P was extracted from whole embryos of the indicated stages and analyzed by LC-ECI-MS/MS. Data are presented as means ± S.D. **, p<0.01. (TIF) [file pone.0094114.s004.tif]

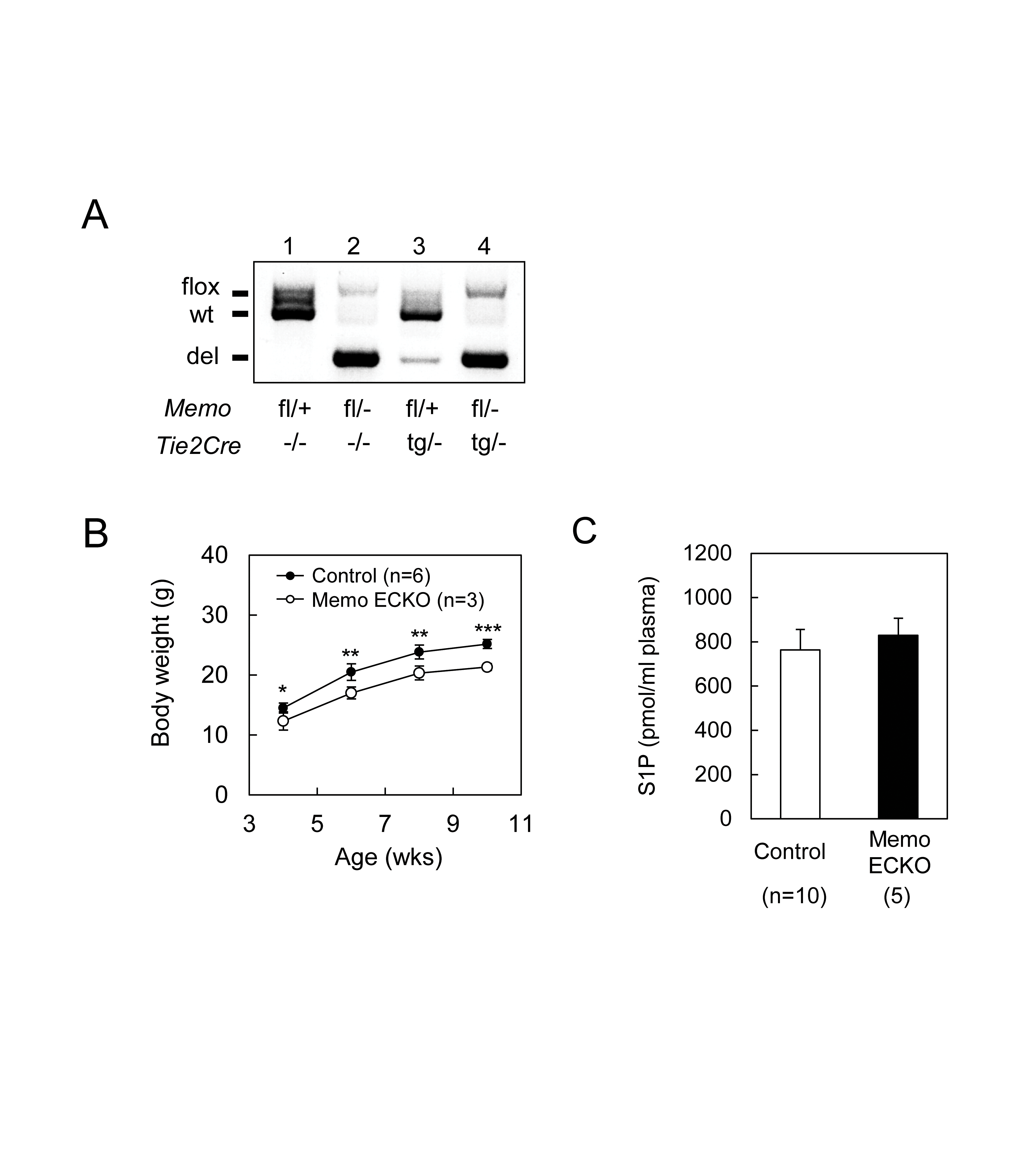

Supplement: Figure S5 — A, Genomic PCR analysis of the Memo gene carried out on ear DNA of the pups at P21. The fragments amplified from the floxed (flox), wild-type (wt) and deleted (del) allele of the Memo gene are indicated. The four possible genotypes i.e. 1) Memo fl/+::Tie2Cre −/−, 2) Memo fl/−::Tie2Cre −/−, 3) Memo fl/+:: Tie2Cre tg/− and 4) Memo fl/−:: Tie2Cre tg/− are represented. B, Body weight of control and Memo ECKO mice. Data are presented as means ± S.E. C, S1P level in control and Memo KO mouse plasma. S1P was extracted from plasma of adult mice (12-weeks old) and analyzed by LC-ECI-MS/MS. Data are presented as means ± S.D. *, p<0.05; **, p<0.01; ***, p<0.001. (TIF) [file pone.0094114.s005.tif]

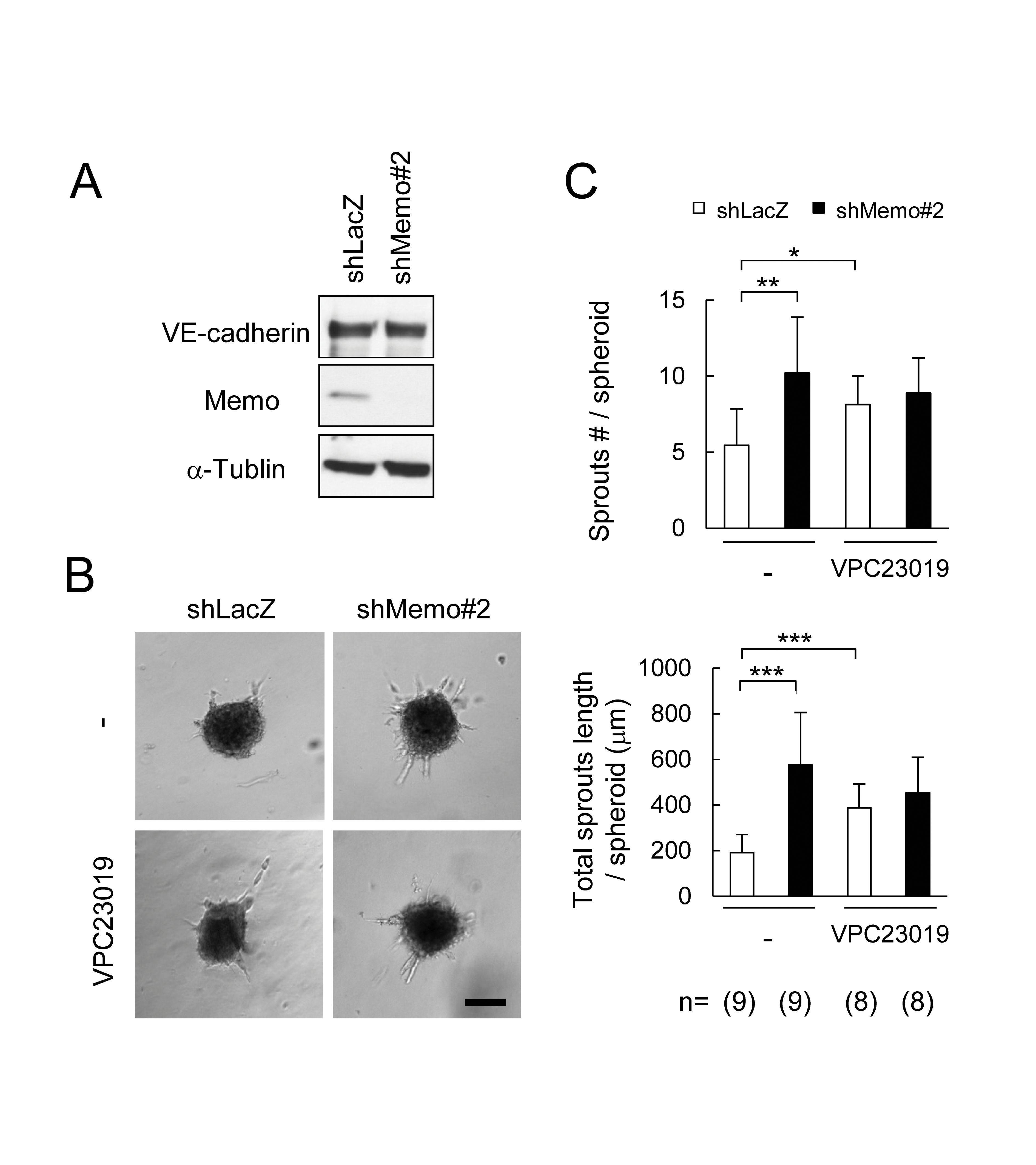

Supplement: Figure S6 — A, Expression of VE-cadherin in control and Memo KD HUVECs after starvation. Monolayers of HUVECs were cultured in serum-free media for 6 h. Cell lysates were prepared and western analyses were performed with the indicated antibodies. B–C, Sprout formation from multicellular spheroids generated from control and Memo KD HUVECs. Sprouting was compared in the absence and presence of VPC23019 (10 μM). Representative images (B) and quantified results (C) are shown. A part of the data shown in Figure 8B and 8C is shown again in order to compare the effect of non-treated shLacZ and shMemo#2 to VPC23019. The numbers of multicellular spheroids used for each condition are indicated at the bottom of (C). Scale bar in (B), 100 μm. Data in (C) are presented as means ± S.D. of the scores for each multicellular spheroid. *, p<0.05; **, p<0.01; ***, p<0.001. (TIF) [file pone.0094114.s006.tif]

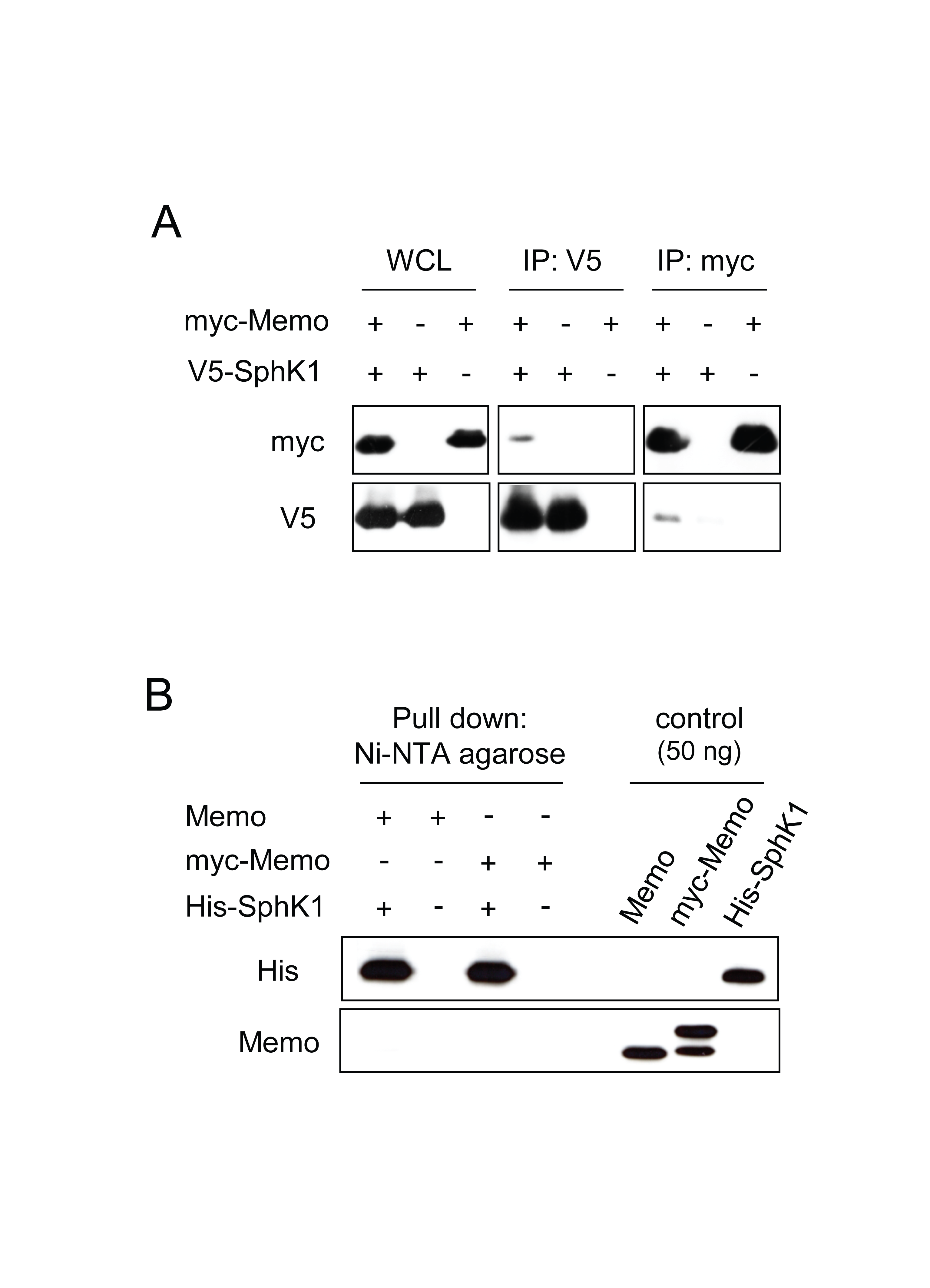

Supplement: Figure S7 — A, Complex formation between Memo and SphK1 in HEK293T cells. Cells were transiently transfected with vectors expressing myc-Memo and/or V5-SphK1. After 48 h, whole-cell lysates (WCL) were prepared and subjected to immunoprecipitation (IP) using either an anti-V5 or anti-myc antibody. Western analyses were performed using the indicated antibodies. B, Recombinant Memo and SphK1 do not directly interact. Recombinant Memo (2 μg) or myc-Memo (2 μg) was incubated with, or without, recombinant His-SphK1 (2 μg) and the protein mixtures were subjected to pull-down assays using Ni-NTA agarose. After washing, the bound proteins were eluted from the agarose and western analyses were performed using the indicated antibodies. 50 ng of each protein was loaded as a control. (TIF) [file pone.0094114.s007.tif]
