# Supplementary material for: Memo Has a Novel Role in S1P Signaling and Crucial for Vascular Development
Source: PLoS One. 2014 Apr 8;9(4):e94114. doi: 10.1371/journal.pone.0094114 (PMC3979765; doi:10.1371/journal.pone.0094114)
Supplement: Table S1 — Primer pairs used for qPCR. (PDF) [file pone.0094114.s008.pdf]

Table S1

Primer pairs used for qPCR

Gene Name	Primer pair
Mouse SphK1	5’-GGAACTTGACTGTCCATACC-3<br>5’-TACCATCAGCTCTCCATCC-3’
Mouse SphK2	5’-GCACGGCGAGTTTGGTTC-3’<br>5’-GAGACCTCATCCAGAGAGACTAG-3’
Mouse S1PR1	5’-TTCTCATCTGCTGCTTCATCATCC-3’<br>5’-GGTCCGAGAGGGCTAGGTTG-3’
Mouse S1PR3	5’-AAGCCTAGCGGGAGAGAAAC-3’<br>5’-TCAGGGAACAATTGGGAGAG-3
Mouse Actin	5’-TGCGTGACATCAAAGAGAAG-3’<br>5’-GATGCCACAGGATTCCATA-3’
Human SphK1	5’-CTTGCAGCTCTTCCGGAGTC-3’<br>5’-GCTCAGTGAGCATCAGCGTG-3’
Human SphK2	5’-CTGTCTGCTCCGAGGACTGC-3’<br>5’-CAAAGGGATTGACCAATAGAAGC-3’
Human S1PR1	5’-TGCGGGAAGGGAGTATGTTT-3’<br>5’-CGATGGCGAGGAGACTGAAC-3’
Human S1PR3	5’-TGATTGTGGTGAGCGTGTTCA-3’<br>5’-GGCCACATCAATGAGGAAGAG-3’
Human GAPDH	5’-GAAGGTGAAGGTCGGAGTC-3’<br>5’-GAAGATGGTGATGGGATTTC-3’
