# Supplementary material for: Memo Has a Novel Role in S1P Signaling and Crucial for Vascular Development
Source: PLoS One. 2014 Apr 8;9(4):e94114. doi: 10.1371/journal.pone.0094114 (PMC3979765; doi:10.1371/journal.pone.0094114)
Supplement: Table S2 — Analysis of embryos from Memo +/− intercrosses. Memo +/− males and females were mated and the genotype of the offspring at the indicated ages was analyzed. The number of embryos with the indicated genotype are listed; the numbers of dead embryos, as judged by the absence of a heartbeat is in the (). * % live Memo −/−/total live embryos. (PDF) [file pone.0094114.s009.pdf]

Table S2

Analysis of embryos from Memo +/- intercrosses

Memo +/- males and females were mated and the genotype of the offspring at the indicated ages was analyzed. The number of embryos with the indicated genotype are listed; the numbers of dead embryos, as judged by the absence of a heartbeat is in the ( ). * % live *Memo* -/- / total live embryos.

Age	*Memo*			% *Memo* -/- *
	+/+	+/-	-/-	
E11.5	4	9	8	38
E12.5	21	85	34	24
E13.5	16 (1)	47	22 (1)	26
E14.5	28	60 (1)	22 (10)	20
E15.5	16	27 (2)	8 (10)	16
E16.5	12	29	2 (4)	5
E17.5	4	20	1 (2)	4
E18.5	9	11	0 (2)	0
